# A quasi-experimental effectiveness evaluation of a nurse-led Hospital Violence Intervention Programme in the United Kingdom using linked routine health and administrative data

**DOI:** 10.1016/j.eclinm.2026.103848

**Published:** 2026-03-26

**Authors:** Simon C. Moore, Sinead Brophy, Amrita Bandyopadhyay, Megan Hamilton, Henry Yeoman, Adele Battaglia, David O'Reilly, Lara Snowdon, Jonathan Shepherd, Danny Tod, Alan Watkins, Vas Sivarajasingam, Simon Walker, Shainur Premji

**Affiliations:** a Violence Research Group, School of Dentistry, Heath Park, Cardiff, CF14 4XY, United Kingdom; b Security, Crime and Intelligence Institute, Cardiff University, PO Box 1182, Cardiff, CF11 1AZ, United Kingdom; c Centre for the Improvement of Population Health Through e-Records Research (CIPHER), Data Science Building, Swansea University, Singleton Park, Swansea, SA2 8PP, United Kingdom; d Cardiff Liver Unit, Cardiff and Vale NHS Trust, Cardiff, United Kingdom; e Academic Department of Military Surgery and Trauma, Defence Medical Services, Birmingham, B15 2SQ, United Kingdom; f World Health Organization Collaborating Centre on Investment for Health and Wellbeing, Policy and International Health, Public Health Wales, Tyndall Street, Cardiff, CF10 4BZ, United Kingdom; g York Trial Unit, Department of Health Sciences, University of York, Heslington, York, YO10 5DD, United Kingdom; h Swansea University Medical School, Swansea University, Singleton Park, Swansea, SA2 8PP, United Kingdom; i Centre for Health Economics, University of York, Heslington, York, YO10 5DD, United Kingdom

**Keywords:** Violence prevention, Emergency department, Public health

## Abstract

**Background:**

Emergency Department (ED) care of people injured in violence is increasingly seen as an opportunity to address patients’ psychosocial vulnerabilities as well as their treatment. These vulnerabilities include alcohol, drug use, mental health and social vulnerabilities that are also associated with high levels of unplanned ED attendances for reasons other than violent injury. Since evidence of the effectiveness of interventions designed to reduce these risks is weak, we evaluated the impact of nurse-led Hospital Violence Intervention Programmes (HVIPs) across two sites in Wales, United Kingdom, on subsequent unplanned ED attendance.

**Methods:**

We used anonymised country-wide electronic health and administrative data to identify a cohort of patients, from 2019 to 2024, injured in violence who attended EDs in Wales, United Kingdom. We matched the characteristics of patients who engaged with the HVIP to control patients in the same cohort. We estimated the unadjusted hazard ratio (HR) for subsequent unplanned ED attendances for patients, and adjusted HRs to determine HVIP effectiveness overall and for sub-groups based on age and gender. This study is registered with ISRCTN (68945844).

**Findings:**

For patients who engaged with the intervention (n = 2068; representing 3580 attendances), the frequency of subsequent ED attendances was lower than control patients (n = 6196; 12,174 attendances; HR = 0.95, 95% CI 0.91–0.99). The intervention was more effective for female patients (HR = 0.86, 95% CI 0.80–0.92) and those aged 11–17 (HR = 0.88, 95% CI 0.82–0.92) and 18 to 30 (HR = 0.86, 95% CI 0.80–0.92) years of age.

**Interpretation:**

If risk factors associated with violence related injury are identified and addressed as part of ED care, ED attendances can be reduced.

**Funding:**

This study is funded by the National Institute for Health and Care Research, Public Health Research Programme (NIHR134055).

**Research in context**

**Evidence before this study**

Hospital Violence Intervention Programmes (HVIPs) in Emergency Departments (EDs) aim to identify patients injured in violence, their violence related vulnerabilities, and to reduce risks of repeat violent injury. Evaluations are largely limited to low-quality controlled trials in the United States and qualitative approaches. HVIPs may reduce violent reinjury but higher quality evaluations are needed to identify the conditions in which they may succeed. We searched PubMed, Web of Knowledge/Science Direct, EBSCOHost (PubMed, CINAHL with Full Text, MEDLINE), Google Scholar, BioMed Central and the World Health Organisation library from database inception to March 2023, without language restrictions, using search terms that primarily included “trial,” “violence” and “emergency department” and variations thereof, 35 papers were retained for full text review.

**Added value of this study**

This study shows that nurse led HVIPs embedded in the ED clinical team can identify patients’ vulnerabilities and by addressing them, through data sharing and referral to selected agencies for specific intervention and advice, subsequent unplanned ED attendance can be reduced significantly, especially among women, girls and those aged under 31 years of age.

**Implications of all the available evidence**

The implications of evidence previously generated, and the new evidence presented here are, first, that people injured in violence can be identified by nurses in EDs; this is important since, globally, at least half of violent incidents are not known to police. Second, individual psychosocial vulnerabilities can also be identified in EDs; these may include criminal and sexual exploitation, modern day slavery and radicalisation as well as known vulnerabilities like alcohol and drug misuse and psychiatric illness. Third, referral and data sharing by nurses to agencies able to address these vulnerabilities—which are also common to other conditions and circumstances in which people seek ED treatment—is practical and effective. Overall, nurse led HVIPs in EDs are, through the identification and mitigation of risks associated with injury in violence, an effective means of reducing ED attendances, especially among women and girls and those aged under 31 years of age.

## Introduction

Emergency Departments (EDs) are ideal locations for Hospital-Based Violence Intervention Programmes (HVIPs), as at least half of those exposed to serious violence and attending ED are unknown to law enforcement.^1^, ^2^, ^3^, ^4^, ^5^, ^6^ HVIPs are a public health response to violence, contributing to patient discharge planning and referral into organisations able to address any underlying psychosocial vulnerabilities associated with violence.^6^^,^^7^ Although ED clinicians in the United Kingdom (UK) are expected to support patients’ psychosocial needs,^8^ opportunities to do so are limited by time constraints and performance targets.^9^ Accordingly, health promotion activities imposed on EDs can receive limited engagement.^8^ The nurse-led HVIPs, considered here as Violence Prevention Teams (VPTs), work within the ED clinical team without imposing an additional burden on ED staff.^9^

Additional HVIP resources may improve the support available to patients for violence-related vulnerabilities including psychiatric illness, neurodevelopmental disorders, criminal and sexual exploitation, drug and alcohol misuse.^9^ HVIPs are further aligned to UK Government policies that promote whole system multi-agency approaches to violence, and the HVIP considered here arose through a collaboration between the National Health Service (NHS) and the South Wales Police and Crime Commissioner.^10^^,^^11^ Although HVIPs have been implemented in the UK, North America and elsewhere, their effectiveness has received limited attention.^12^ Existing research on the effectiveness of HVIPs is mainly from North America, where the operationalisation of HVIPs varies. For example, some only accept English speaking patients or young people, some are additional to usual care in ED, whereas others are embedded within the ED clinical team with staff having access to patient records.^12^^,^^13^

This evaluation was undertaken from the perspective of the NHS. As vulnerabilities that increase exposure to violence such as psychiatric illness, drug and alcohol use are also associated with greater ED attendance, the hypothesis was that HVIPs would reduce subsequent unplanned ED attendance, the principal NHS service cost, compared to matched control patients.

## Methods

### Intervention and exposure

The VPTs were implemented at two sites in South Wales (Morriston Hospital ED, Swansea, and University Hospital of Wales ED, Cardiff) to address a perceived under-provision for patients attending due to violence aged 11 years and older. The initial 11–25 years age range matched the available community referral options. However, the upper limit was dropped soon after implementation consistent with the NHS obligation for equitable healthcare. The VPTs were additional to existing resources for domestic and sexual violence (online supplement 1). The VPT was part of the clinical team, nurses were familiar with their local ED and were trained to support patients and able to address patients' reluctance to disclose (patients can be accompanied by the perpetrator, they might distrust any statutory services, fear repercussions, or may regard violence as a normal part of life that has no recourse).^8^^,^^14^ Patients would be referred into the VPT by the broader clinical team. Nurses also read attending patients’ records and approached those who they suspected might have been exposed to violence. Contact with patients was preferably face-to-face and at a time appropriate with regards their treatment needs. However, patients attending when nurses were not on shift could be also contacted by telephone. The nurses identified any psychosocial vulnerabilities and modifiable risks through scrutiny of community and hospital patient records and in conversation with patients and caregivers as appropriate. To support discharge planning they curated a referral network that included statutory (mental health, drug and alcohol, social services, child criminal and sexual exploitation, law enforcement), third sector (victim support, child and adult service), and could refer into the Prevent counter radicalisation programme^15^ and into services supporting those subject to modern day slavery^16^ (online supplement 2). In Cardiff, the nurses could hold a case and provide ongoing support. Both VPTs, with the permission of patients, could share patient details with any service they were referred into. The Template for Intervention Description and Replication (TIDieR) is available in Supplement 1 and an accessible Intervention Manual is available online.^17^

### Study design and population

A controlled longitudinal natural experiment followed a preregistered (ISRCTN 68945844) and published protocol and analytic strategy.^18^ The aim was to determine the effectiveness of the VPTs on subsequent unplanned ED attendances.

The primary hypothesis, reported here, was that VPTs reduce subsequent unplanned ED attendance, compared to matched control patients. Additional exploratory analyses assessed effectiveness for subgroups. A cost-effectiveness analysis is reported separately. The primary analysis population was on a per-protocol (PP) basis defined as eligible patients who had engaged with the intervention and accepted support. The secondary analysis population was on an intention-to-treat (ITT) basis where exposure was attendance in an intervention ED due to violence, irrespective of their engagement with the intervention. Stakeholders requested analysis of an additional subpopulations: patients who engaged with the intervention and were referred (PP_r_) into other services, and additional analysis by intervention site.

The datasets used in the evaluation included whole population routine health and demographic records for all living residents of Wales, UK, identified through registrations with a General Practitioner (GP). These records were accessed in the Secure Anonymised Information Linkage (SAIL) databank, an ISO 27001 certified and UK Statistics Authority accredited secure data environment (online supplement 3).^19^ Each resident in Wales were assigned an encrypted anonymised linkage field (ALF), either derived directly from their NHS number or by using approximate string matching to their sex, date of birth and place of residence. Only patients with an identified ALF or greater than 90% certainty of a matching ALF, in the case of approximate matching, and included in the Welsh Demographic Service (WDS) dataset were included in the study cohort. The WDS also included dates of residence in Lower Layer Super Output Areas (LSOAs, small areas typically containing 400 to 1200 households), from which areas descriptors including deprivation and rurality were available, derived at the time of their first residence in Wales after November 2019.^20^ ALFs enable individual linkage across the WDS to datasets in SAIL, including the Emergency Department Dataset (EDDS), Annual Death Data Extract (ADDE), patient episode database Wales (PEDW, dates of admission into hospital including diagnostic codes), and the Welsh Longitudinal General Practitioner (WLGP) Dataset. Patient ethnicity was retrieved from the 2021 and 2011 Census at the time of their entry into the cohort^21^; if missing in both then codes were identified from, in order, WLGP, PEDW, and the EDDS. All the datasets are included in the Health Data Research UK Gateway.^22^ The WLGP covers 86% of the population of Wales and 83% of GP practices in Wales. The WDSD is complete coverage of all people registered with a GP in Wales. PEDW and EDDS give complete coverage of all hospital admissions and ED attendances, respectively. Operational data for the first VPT in Cardiff was available from November 2019 until December 2023, and from January 2022 to February 2024 in Swansea. The VPT teams collected data on patients they had identified as attending due to violence and their extent of engagement with the intervention.

### Informed consent

The analyses were undertaken on anonymised data and therefore informed consent from participants was not required.

### Eligibility

Patients were eligible if they had attended an ED due to violence, were residents of Wales, had a corresponding ALF in the WDS and were 11 years of age or older. Patients were ineligible if they were under 11 years of age.

### Intervention patients

Intervention patients were included in the cohort if eligible through either identification in the VPT collected data or intervention ED as having a violence-related attendance during the intervention periods.

### Control patients

Control patients were included in the cohort if eligible and had a violence-related attendance on or after 1st November 2019 in a non-intervention ED recorded in the EDDS.

### Matching

Intersectional risks are likely an important determinant of violence and therefore to minimise residual imbalance we undertook coarsened exact matching (CEM).^23^ Control and intervention patient characteristics used in CEM^24^^,^^25^ were sex, age, ethnicity, Welsh Index of Multiple Deprivation (WIMD) quintile, urban or rural residence, date of inclusion in the cohort, and whether they were admitted into hospital following their initial attendance as a proxy to acuity. Patients were also matched on the number of ED attendances in the preceding month to adjust for unobserved heterogeneity.^26^ Matching was repeated for each primary, secondary and sensitivity analysis. The CEM ratio for the primary analyses was one-to-many (1:M, with M representing all available control patients), with sensitivity analyses using one-to-one (1:1). Further sensitivity analyses used Propensity Score Matching (PSM) using 1:1 and 1:M ratios (online supplement 4).^27^

### Outcomes

The outcome was defined from the perspective of the NHS as the frequency of unplanned ED attendances following entry into the cohort. The characteristics associated with violence are also associated with more frequent unplanned ED attendance.^28^ If identified and support is provided to patients then the expectation is less frequent ED attendances.^29^, ^30^, ^31^

### Statistical analysis

Cohort patients’ subsequent unplanned ED attendances were recurrent events, independent and unordered. The time series was right-side censored by death, moving residence outside of Wales, and date that EDDS records were available until (July 2024) (online supplement 5). For serious trauma, patients can be transferred across hospitals once admitted resulting in multiple and therefore subordinate ED attendances that are not independent. Time at risk was therefore interval censored by spells of admission into hospital.^32^^,^^33^ We implemented CEM weighted recurrent event analysis to determine the hazard ratio (HR) by allocation.^26^ Sample size estimates indicated a study arm sample size of 300 would be sufficient to detect a significant effect (at α = 0.05, β = 0.90).^18^ The primary analysis was PP CEM 1:M across both intervention sites, treatment was additionally interacted with sex, age group (11–17, 18–30, 31–50, and 50+ years), ethnicity, WIMD quintile, urban or rural residence, admission into hospital following ED attendance, and a binary variable describing no (0) or one or more (1) prior month ED attendances. Derivation of the study data sets is available in online supplement 6. Data cleaning was undertaken using Structured Query Language (SQL) in an IBM Db2 database. Matching and analyses was undertaken in Stata V18.^34^ A fully adjusted PP model further considered interactions between patient characteristics (age, gender, residential characteristics, ethnicity, acuity) and Stata v18 lincom was used to estimate the HR on specific treatment group by characteristic interactions.

### Public and Patient Involvement

The objectives, development and methodology of the overall study was informed through Public and Patient Involvement (PPI), facilitated by two lay members on the project team (HY and AB), and further engagement with those having relevant lived or living experience.^18^ Their experiences captured additional insights, personal perspectives, informed the study design and the interpretation of results (online supplement 7).

### Ethics and permissions

Approval to access and analyse data housed in the databank was granted following independent review by the SAIL Independent Information Governance Review Panel (approval number 1421).

### Reporting

This study is reported in accordance with the Reporting of Studies Conducted using Observational Routinely Collected Data guidelines (STROBE; online supplement 8).

### Role of the funding source

The funder had no role in the design and conduct of the study; collection, management, analysis, and interpretation of the data; preparation, review, or approval of the manuscript; and decision to submit the manuscript for publication.

## Results

### Patient population

Table 1 presents descriptive statistics for the PP 1:M cohort. For the PP analysis, 2068 VPT patients could be matched to 6196 control patients and, for the ITT analysis, 5963 VPT patients could be matched to 17,919 control patients. VPT records suggest 12% of patients were on probation, 37% had mental health needs. Other vulnerabilities included autism spectrum disorder, ADHD, learning difficulties, and homelessness. Furthermore, 31% were associated with weapon-related violence, a police report was made for 50%; 6% were of school age and referred to a School Nurse; a multi-agency referral was made for 19%; 5% were referred to their social worker; 28% were referred into mental health services. Other referrals included to the Prevent counter radicalisation programme,^15^ and support for Modern Day Slavery.^16^

**Table 1:** Descriptive statistics for the CEM 1:M control and treatment groups.

Characteristic	Control (n = 6196)		Treatment (n = 2068)	
	n	%	n	%
Sex				
Female	1844	29.76%	545	26.35%
Male	4352	70.24%	1523	73.65%
Age				
Age 11–17 years	1372	22.14%	651	31.48%
Age 18–30 years	2500	40.35%	695	33.61%
Age 31–50 years	1919	30.97%	550	26.60%
Age 50+ years	405	6.54%	172	8.32%
Ethnic group				
White: English, Welsh, Scottish, Northern Irish or British	5586	90.15%	1562	75.53%
Mixed or Multiple	37	0.60%	35	1.69%
Asian: Asian British or Asian Welsh, Bangladeshi	108	1.74%	106	5.13%
Black: Black British, Black Welsh, Caribbean or African, African	22	0.36%	17	0.82%
Other	424	6.84%	335	16.20%
Missing	19	0.31%	13	0.63%
Discharge destination				
Discharged home	6139	99.08%	2030	98.16%
Admitted into hospital	57	0.92%	38	1.84%
Residence^a^				
Rural	318	5.13%	107	5.17%
Urban	5878	94.87%	1961	94.83%
Deprivation				
Most deprived quintile	2772	44.74%	963	46.57%
2nd quintile	1640	26.47%	367	17.75%
3rd quintile	801	12.93%	274	13.25%
4th quintile	547	8.83%	215	10.40%
Least deprived quintile	436	7.04%	249	12.04%
Previous month ED attendance^b^				
No ED attendance	6064	97.87%	1958	94.68%
One or more ED attendances	132	2.13%	110	5.32%

a Rural includes rural town and fringe, rural town and fringe in a sparse setting, rural village and dispersed, rural village and dispersed in a sparse setting; urban includes urban city and town, urban city and town in a sparse setting.

b Previous ED attendance was 0, 1, 2, 3, 4, 5+ in the main analysis, reduced to a binary 0, 1 indicator in subsequent interactions and presented here as such to mitigate risk of disclosure.

### Event analysis

Primary analyses (PP, Table 2) indicate that the intervention was associated with a significant reduction in the frequency of subsequent ED attendances of approximately 5%. This generalised to all eligible patients in an intervention ED, with an overall reduction of 3%. No effect was observed for patients engaging with the intervention and who were referred into other services.

**Table 2:** CEM weighted recurrent event analysis, with weighted and unweighted number of patients and corresponding attendances.

	HR	95% CI	Unweighted				Weighted			
			Patients, n		ED attendances, n		Patients, n		ED attendances, n	
			Control	Intervention	Control	Intervention	Control	Intervention	Control	Intervention
PP	0.95	0.91–0.99	6196	2068	12,174	3580	2068.00	6196.00	11,432.17	3580.00
PP_r_	0.99	0.94–1.04	5142	1226	9699	2096	1226.00	5142.00	8980.01	2096.00
ITT	0.97	0.94–0.99	8926	5963	17,919	10,304	5963.00	8926.25	16,097.72	10,304.00

Sensitivity analyses (online supplement 9) were broadly consistent with the results presented in Table 2, except for the PSM 1:M (HR = 0.97, 95% CI 0.94–1.01), which may be attributable to difference in PSM and CEM algorithms with the latter better accounting for intersectional characteristics of patients.

A fully adjusted model, replicating the weighted CEM analysis for the PP group, including variables used in matching (online supplement 10), with each interacted with treatment group, explored the effectiveness of the intervention for sub-groups. The overall HR fell (HR = 0.58, 95% CI 0.46–0.73), and interactions of note (Table 3) suggest that the intervention was more likely to improve outcomes for female patients, those aged 11–30 years of age or 18–30 years, and those with a previous months ED attendance. Severe outcome analyses considered the survival until death for all patients included in the primary analysis. Due to low numbers, disclosure rules prevent the presentation of these data. There were no significant differences in survival by allocation and for death attributable to violence.

**Table 3:** Estimated effects of the intervention on hazard ratios for age, gender and previous month ED attendance.

	HR	95% CI
Gender		
Female	0.86	0.80–0.92
Male	1.04	0.99–1.08
Age (years)		
Age 11–17	0.88	0.82–0.92
Age 18–30	0.86	0.80–0.92
Age 31–50	1.19	1.12–1.27
Age 50+	1.17	1.07–1.35
Previous Month Attendance		
None	1.02	0.98–1.06
One or more	0.77	0.69–0.86

## Discussion

Nurse-led VPTs reduced the frequency of subsequent unplanned ED attendances for patients attending due to violence, compared to matched control patients. These results are broadly consistent with studies in North America where HVIPs are effective across outcomes including violence recidivism and arrests for violence, an earlier process and implementation evaluation, and analysis demonstrating VPTs were able to overcome patients’ barriers to disclosure associated with age, sex, ethnicity and residential deprivation.^9^^,^^13^, ^14^ Our process evaluation found VPT nurses engaged with members of the ED clinical team and provided ongoing training to new and agency staff. The perception was that these activities improved engagement with vulnerable patients generally, potentially explaining the VPT effect for all patients attending ED because of violence found here.^9^^,^^13^ Furthermore, a barrier in provisioning support is non-disclosure, reasons for which can include a patient accompanied by the perpetrator, a distrust of authority that generalises ED staff, and those from ethnic minority groups who may experience discrimination that further erodes trust.^14^ Regarding violence, the VPTs are likely to improve the health of patients, reduce reattendance and address safeguarding inequalities associated with protected characteristics. Further analysis suggested that the effect on ED attendance was strongest for women and those who were 31 years of age or younger, suggesting that the VPTs may contribute to initiatives concerning violence against women and girls, and high intensity users.^28^, ^35^ However, analyses also suggested a relative increase in ED attendances in the 31 years of age and older groups, compared to younger patients. This suggests that the overall effect of the intervention varies by patient characteristic and there is therefore a need to better understand how the intervention might be refined to better deliver more equitable support.

The research literature on behaviour change across the life course is limited, and especially for violence prevention where greatest attention is paid to younger cohorts. The results for older cohorts are interpreted in the context of UK policy where violence prevention in younger cohorts is emphasised, and therefore a relative scarcity of services aimed at supporting older patients. Moreover, those factors associated with a greater exposure to violence and ED attendance generally (alcohol, illicit substance misuse, and psychaitric ill health), might be more ingrained and therefore less tractable in older cohorts. Furthermore, exposure to violence in older generations may be attributable to factors beyond alcohol, illicit substance misuse, and psychiatric illness for which the intervention may have been less able to provision appropriate support. VPTs that build trust and encourage engagement might therefore increase older patients’ willingness to engage with ED but have fewer opportunities to find the support they need. The intervention was less effective for those who had been referred into other services. This is may be attributable to greater acuity and therefore a requirement for specialist care. For example, acute mental health referrals can be subject to long waiting lists, potentially minimising the effect across the timeline considered here. The effect of waiting lists can be considered with a longer follow-up period and greater granularity.^36^

More generally, there is paucity of knowledge on the characteristics of patients attending ED due to violence, the pathways they follow before, during and after their time in ED, and the extent that the VPTs can identify patients attending due to violence who were otherwise missed under routine care. During analyses undertaken for this evaluation, it became apparent that in future research greater attention should be paid to what constitutes a credible population for analysis.

Further research will consider the cost-effectiveness of VPTs and the extent they benefit patient health. While VPTs have access to a range of community and hospital data systems containing patient records, it is unlikely they identify undiagnosed psychosocial vulnerabilities. Future research might consider how VPTs can improve the identification of, for example, psychiatric illness and neurodevelopmental conditions, and therefore better consider the mechanisms of action behind the results reported here.^29^ Moreover, given the lessened impact on male and older patients, tractable solutions that enhance support for these groups should be assessed, and especially for those involved in the criminal justice system. Finally, given the waiting lists for specialist care and the association of violence with deprivation, consideration might be given to prioritising referrals for specific patient groups exposed to violence.^36^^,^^37^ Support given to patients by the VPTs, patient referral to external organisation able to provide specialist support, and nurses' ability to build trust with patients are the presumed mechanisms of action. However, this may not be the case for all patients. Our PPI engagement suggested that there are some patents who simply do not believe that the violence they experience is anything but normal, some refuse support as they are fearful of losing their children, other reasons are related to ethnicity, and age (young men may prefer to maintain their masculine persona).^14^ Further work should undertake interviews with a range of patients to better understand how the intervention worked or did not. Finally, the generalisability of these results should be considered. It is feasible population characteristics may influence the nature of violence in an ED's catchment, and therefore the nature of services required to support patients.^13^

As with all studies using routinely linked data, only those who can be identified in routine data and linked across datasets can be included. This may exclude certain groups, who may wish to avoid contact with statutory services. Furthermore, some patient groups may avoid ED or avoid being identified in ED and would not therefore be included here. Our use of routine health data means attrition does not affect the analysis, however during the overall project we found that VPTs identified patients attending due to violence who were not identified as such in routine ED data.^14^ Although this likely represents an improvement to patient care, it may also be feasible that the reported effects are partly attributable to patient self-selection into the intervention (online supplement 9, Table S35). Factors associated with patient non-disclosure under routine care include ethnicity, age, gender, and residential deprivation, which were used in matching. It is possible that other unmeasured factors mean that there remains imbalance across control and intervention groups. Our findings also suggest that those identified in the treatment areas had a greater number of past ED attendances. As past ED attendance predicts future ED attendance, this likely biases the analyses towards the null. It is possible that the treatment areas could theoretically have identified those with lower risk of future ED attendance by seeking out those who have experienced violence but not disclosed that under routine care, who were subsequently included in the treatment groups. However, we did not find any evidence of this. Without an objective measure of violence, that does not rely on self-report, it is not possible to fully ascertain the influence of non-disclosure. This might be partly mitigated in future through additional linkage with police and other datasets that record violence. As the intervention will continue for a longer period, we anticipate undertaking additional analyses alongside matching to assess any site-level differences to assess the influence of local and national policies.

In conclusion, this study shows that nurse led HVIPs embedded in the ED clinical team can identify patients injured in violence and their psychosocial vulnerabilities. Through referral into selected agencies for interventions and support, and sharing patient details with those agencies, subsequent unplanned ED attendances are significantly reduced, especially among women, girls and those aged under 31 years of age.

## Contributors

SCM, SB, AB, HY, AB, DO'R, LS, JPS, VS, AW, SW conceived this study. SCM, AB, DT, AW, SW, SP drafted the statistical analysis plan. AB, JPS, DT, AW, SP led on the analysis. SCM, MH, JPS, DT, AW, SW, SP drafted the manuscript. SCM, SB, AB coordinated approvals for and access to data within SAIL. SCM, SB, AB, MH, HY, AB, DO'R, LS, JPS, DT, VS, AW, SW, SP edited later versions of the manuscript. SCM, AB, DT, AW, SW, SP had full access to and verified the data and analytics. SCM was project lead and took responsibility for the final decision to submit the manuscript.

## Data sharing statement

As the data used in this study include private medical information it cannot be shared. However, researchers can apply for access to both data and the analytic code used from SAIL (www.saildatabank.com).

## Declaration of interests

SM reported receiving grants from the Medical Research Council (MRC), National Institute for Health and Care Research (NIHR), Youth Endowment Fund, Office of the South Wales Police and Crime Commissioner's Office, outside the submitted work. SM is Deputy Chair of the NIHR Better Methods Better Research Funding Panel and received honoraria as Visiting Professor from Università Cattolica Del Sacro Cuore, Milan, Italy. SB reported receiving grants from NIHR and sits on the NIHR Public Health Research Board. SW reported receiving grants from the NIHR. The institutions affiliated with SM, SB, HY, AB, DO’R, LS, JS, AW, VS and SW all received funds for the current study. No other competing interests were declared.

## Appendix A Supplementary data

Supplements

EDVIPE_protocol
