# Supplementary material for: A quasi-experimental effectiveness evaluation of a nurse-led Hospital Violence Intervention Programme in the United Kingdom using linked routine health and administrative data
Source: eClinicalMedicine. 2026 Mar 26;94:103848. doi: 10.1016/j.eclinm.2026.103848 (PMC13054530; doi:10.1016/j.eclinm.2026.103848)
Supplement: Supplements [file mmc1.docx]

**Supplementary Appendix**

**A Quasi-Experimental Effectiveness Evaluation of a Nurse-led Hospital Violence Intervention Programme in the United Kingdom**

**Contents**

[Supplement 1: TIDieR 2](#_Toc221779428)

[Supplement 2: Violence Prevention Team Referral Network 7](#_Toc221779429)

[Supplement 3: Study Data Sets 10](#_Toc221779430)

[Data Tables 10](#_Toc221779431)

[Derived Variables 11](#_Toc221779432)

[Supplement 4: Matching 12](#_Toc221779433)

[Coarsening 12](#_Toc221779434)

[Variables included in Coarsened Exact Matching 13](#_Toc221779435)

[Supplement 5: Time Series and Discontinuous Risk 14](#_Toc221779436)

[Supplement 6: Data Derivation 15](#_Toc221779437)

[Data flow diagram for Coarsened Exact Matching (CEM) 15](#_Toc221779438)

[Outcome Data 16](#_Toc221779439)

[Supplement 7: Patient and Public Involvement 23](#_Toc221779440)

[Reflections 24](#_Toc221779441)

[Conclusion 25](#_Toc221779442)

[Supplement 8: RECORD and STROBE checklist 26](#_Toc221779443)

[Supplement 9: Primary, secondary and sensitivity analyses 33](#_Toc221779444)

[Supplement 10: Interactions 36](#_Toc221779445)

[References 39](#_Toc221779446)

# Supplement 1: TIDieR

The Template for Intervention Description and Replication (TIDieR) is used to describe the HVIPs evaluated, which was adapted from a formative evaluation of the Cardiff Violence Prevention Team. ^1 2^

### Brief Name

Violence Prevention Team (VPT)

### Preparation of TIDieR

Preparation of TIDieR was undertaken by

- Cardiff University (Violence Research Group and DECIPHER)
- Youth Endowment Fund (YEF), who also part-funded the intervention and funded the process and implementation evaluation.
- An expert advisory group provided oversight, including experts from Public Health Wales, Welsh Trauma Network, South Wales Police, Office of the Police and Crime Commissioner, and third sector organisations including Welsh Women’s Aid, The Wallich and Black Association of Women Step Out (BAWSO).

### Service Delivery

- VPT staff (nurse, nurse advocate, community youth workers), who implemented and continually developed the service.
- Clinical teams in the two Emergency Departments (EDs) in which the interventions are situated provide clinical governance.

### Commissioners

- Wales VPU (partner agencies including South Wales Police Office for the Police and Crime Commissioner and Public Health Wales) provided the bulk of the funding for the interventions and have on-going dialogues regarding the service development, implementation, and delivery at both implementation sites.
- The UK Home Office provided initial funding to the VPU and YEF.

### Intervention Need

EDs receive for treatment patients who have been exposed to violence. There are numerous reasons why some people are exposed to violence, including their alcohol use, illicit drug use, childhood adversity, relationship violence, coercion or exploitation, amongst other reasons. These risk factors will also be associated with increased utilisation of emergency care generally. EDs therefore have a unique opportunity to offer these patients additional support, beyond treatment for acute health needs, either directly or through referral to other healthcare services, or in discharge planning signposting third-sector organisations, for example. If this support is successful, then the expectation is that patients will exhibit a reduced use of emergency care services in general, not just for violence-related injury.

The primary objectives of the hospital-based service provision are to

1. identify patients attending EDs whose attendance is predicated on their exposure to violence,
2. work with patients to understand any circumstances or risk factors that increase their exposure to violence, and
3. to either support their referral into secondary or third-sector care or to provide ongoing case-management alongside third-sector support.

All patient-facing clinical staff in emergency care, and elsewhere, have a duty of care and will have received training necessary to undertake patient safeguarding. However, the additional resources involved with the VPT afford

1. greater time working with patients to determine need,
2. deepen links with primary, secondary, and tertiary care, and third sector organisations, for improved referral processes,
3. work across clinical teams to support colleagues with their safeguarding needs and referral into the intervention, and
4. through direct contact with patients, support opportunities for disclosure and therefore increase ascertainment of assault-related attendances.

While the VPT can and will refer to any community service provider based on patient need, specific funding has been dedicated to two different organisations where the VPTs are located. The community-based provision provides intensive support to high-risk children and young people (aged 11-24 years) involved in serious organised crime, drug-related activity, and showing signs of exploitation. The objective is to build resilience to enable these young people to be diverted away from further involvement in serious violence and organised crime.

### Physical or Informational Materials Used in the Intervention.

The VPTs have made resources available to patients and their families, including leaflets providing details of the service, and information packs to inform them of key issues (e.g., county lines and exploitation).

The VPT has available physical and informational materials usually available to ED staff. This includes access to patient records, both specific to the ED and community healthcare generally. This information facilitates risk assessment and safeguarding practices (e.g., identify patterns of attendance at health care settings for violent-related injury), as well as allowing the VPT ensure their engagement with patients is appropriate at that time (with consideration to the patient’s clinical needs).

In addition, the VPT have strong links with South Wales Police, and receive information and intelligence relating to violence related incidents and community problems. The provision of this information and intelligence to the intervention teams can help inform their interactions with patients and ensure the safety of the hospital and patients (e.g., in cases of youth violence where further attempts to harm a patient may occur).

The VPT provide training to a wide range of clinical and non-clinical staff within the hospital, including

- Clinical hospital staff, on violence (e.g., youth violence) and vulnerability, identifying violence-related injury and engaging with patients, implementing safeguarding procedures for violence-related injuries (e.g., completing multi-agency referral forms, MARFs).
- Reception staff, on data entry and patient coding, to improve the quality of routine clinical data.
- The nature of medical education entails short-term postgraduate and specialty training, and therefore a steady flow of clinical staff new to the ED environment. The VPT provides education and training, and impromptu advice and support through one-on-one interactions, to hospital staff. This includes providing consultation on patients and facilitating patient interactions for more challenging cases.

### The procedures, activities, and/or processes used in the intervention, including any enabling or support activities.

The VPT engages with patients at different stages of their journey, depending on the type and severity of their injuries, the time and day they attend the ED, and the longevity of their hospital stay. They are embedded in the ED clinical team, and have the same resources (e.g., access to electronic patient management systems) as other ED staff.

Referrals: The VPT can receive patient referrals through multiple channels, including email, phone, and face-to-face contact. During their shift, VPT staff can be notified of eligible patients through a set of questions asked during patient registration, triage or by monitoring the ED patient management system. When the VPT is not on shift, paper-based referral forms are available, and staff can still use written formats to refer patients and the VPT will retrospectively review the ED patient management system to identify any additional patients that may have been missed by clinical staff.

Processes: The VPT provides individualised support to patients based on their needs and collaborates with primary, secondary and tertiary care, third sector organisations and other statutory organisations (e.g., local government safeguarding teams, the police, and school nurses). They establish a relationship with the patient and, where appropriate, their family. They provide emotional and practical support. They also manage risk factors and risks to patients by gathering information and through collaborative multiagency work. This can be through existing resources, such as the Domestic Abuse, Stalking, Harassment and Honour Based Violence Assessment (DASH) tool, and referrals into the Multi Agency Risk Assessment Committee (MARAC).

The VPT will either discharge patients to receive support elsewhere, or, in the case of high risk, high need children exposed to criminal or sexual exploitation, continue their support of the patient alongside third sector organisations specifically funded to support the VPT in this area.

### Access to Information

The VPT has access to usual healthcare patient management systems, both ED specific and community-based, that can be accessed to inform patient assessments and safeguarding decisions.

Furthermore, the VPT access information on patients to inform risk assessment and management. For example, following incidents of serious violence (e.g., stabbings and shootings), they will gather information on risks and known associations through the police, probation, hospital staff, and through the safeguarding team (who routinely meet to discuss patients).

### Assessment

The VPT completes a risk- and needs-based assessment in collaboration with patients if they agree to engage and, where appropriate, with their families. This will also include information obtained through patient records (e.g., PARIS and clinical portal) on previous hospital visits.

### Expertise, Background and any Specific Training Given to Each Intervention Provider.

VPT staff will be trained to Level 2 or Level 3 Safeguarding, available through continuing professional development to all frontline staff in the NHS. VPT staff are typically seconded from the broader ED team and will typically be nurse-led.

Additional third-sector support is funded to provide support to high risk, high need children.

### Modes of Delivery

The VPT team meets with patients face-to-face in the ED if they are on shift, or on hospital wards if the patient is admitted. If the team is not on shift, or receive referrals from Minor Injury Units, they will follow-up with a phone call and conduct an assessment. All contact is done an individual basis, or in collaboration with the family (for under 16-year-olds, or in cases the patient consents to family involvement).

Under usual safeguarding processes, the clinician who first receives any disclosure is expected to lead on the subsequent referral to the VPT, so that patients are not required to repeatedly describe circumstances they might find distressing. In this latter case, the VPT will support the patient’s referral.

### Location of the Intervention

The VPT is physically based in the ED. However, they can accept referrals from Minor Injury Units and also provide support to patients on the wards if they are admitted following serious injury. The VPT also works with patients that are transferred from other health care facilities, which is particularly pertinent when the hospital is a major trauma centre, or trauma unit, and therefore accepts out-of-area patients. The VPT engage with other health care settings to facilitate transfers (e.g., provide information on known risks and risk factors), and inform care planning.

### Intervention delivery, number of sessions, schedule, duration, intensity, and dose.

Patients will either engage with the VPT or will refuse support. If the latter, they will have been in contact with the VPT once. For patients who engage with the VPT, the frequency and duration of engagement with patients will be determined through the patient’s age, clinical need, and any underlying vulnerability.

### Hospital-based VPT

The VPT typically has one or two interactions with most patients, such as a phone call or text message. However, patients with greater needs may have more frequent contact and remain on the caseload for several weeks. Inpatients are supported until they are discharged. The VPT can only maintain a small caseload of patients who require longer-term support, typically those on waiting lists or who are too vulnerable to disengage with. Patients referred to the caseworker have minimal involvement with hospital-based services.

### Caseworker

The caseworker offers high-intensity support for high-risk, high-need young people and engages with them two to three times a week. To maintain this level of contact, the caseload is limited to five young people at a time. Some service users may be on the caseload for an extended period if they resist engagement. However, for patients referred to hospital-based services, the caseworker has minimal involvement or contact.

### Intervention Tailoring

#### Level of Harm

The VPT engages with patients attending ED in consequence of their exposure to violence. All patients are eligible, and initial assessment will determine how patients are managed. In the case of domestic abuse, the patient will be handed over to an Independent Domestic Violence Advocate (IDVA). In the case of sexual abuse, the patient will be handed over to the Independent Sexual Abuse Advocate or the Sexual Assault Referral Clinic. In the case of self-harm, the patients will be handed over Mental Health Services. The VPT will therefore typically engage with patients attending with non-domestic violence-related injuries, of varying acuity. The team offers immediate support to patients with non-life-threatening injuries, while for high acuity patients, they wait until the patient is stable before offering their services. The VPT team conducts assessments for patients admitted into the hospital, which allows them to provide more intensive support.

#### Caseworker

The caseworker engages with patients either in the hospital or in community settings and maintains a caseload of up to five young people at a time for high intensity support.

### Stage of Implementation

This is an initial TIDieR, generic to two sites at which the VPT model has been implemented. The VPTs at the two sites are at different stages of implementation with one having a significantly longer operational period.

### Assessment of Intervention Fidelity

The VPT model is subject to a formal evaluation, and a process and implementation evaluation, which this TIDieR informs. The outcome from these evaluations will be a revised logic model, an understanding of the adaptations made to the intervention based on locality, and a formal effectiveness and cost-effectiveness evaluation.

### Actual Intervention Fidelity

Delivery of the intervention has been impacted by staffing changes in both sites, with both sites operating with reduced staff during periods of their operation. As a result, this has led to delays engaging with and referring some patients for further support.

# Supplement 2: Violence Prevention Team Referral Network

The VPTs recorded the immediate destination of patients referred into statutory and third sector services. These referrals are depicted in the network chart in figure S1, showing the Cardiff VPT (CVPT) and Swansea VPT (SVPT). Further information for each destination is provided in table S1.


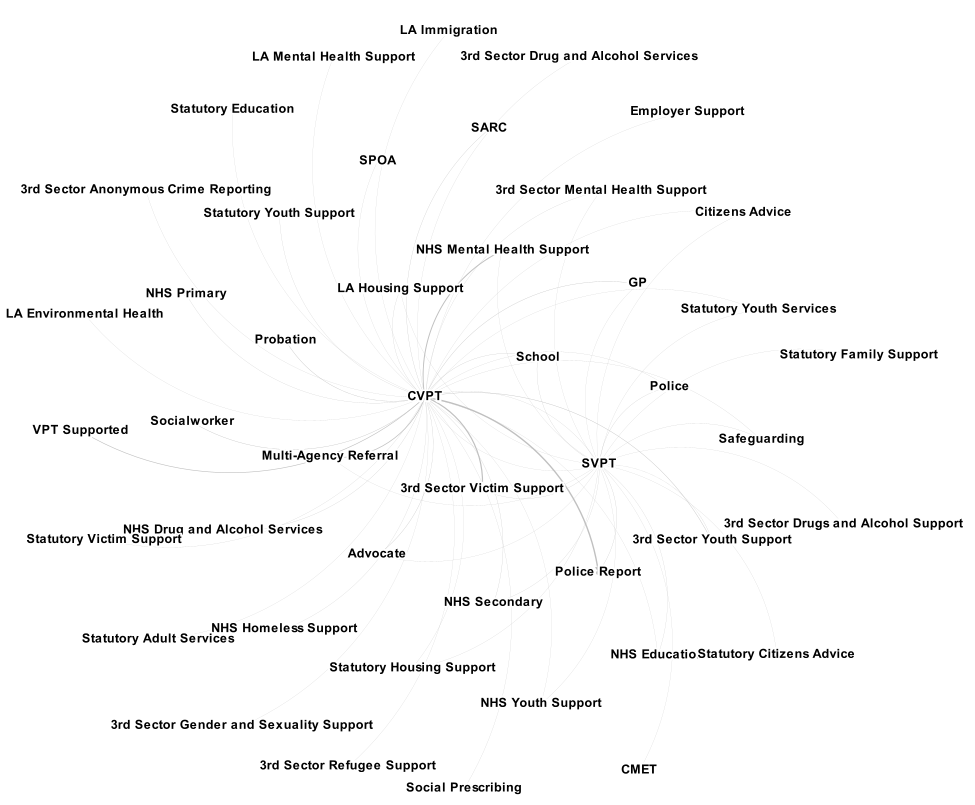


Figure S1 – VPT referral network

Over time, the Violence Prevention Teams curated extensive referral networks through which they were able to refer patients for additional support.

Table S1 – Description of nodes depicted in figure S1

| **Label** | **Description** |
| --- | --- |
| CVPT | Cardiff Violence Prevention Team |
| SVPT | Swansea Violence Prevention Team |
| LA immigration | Immigration support from local authorities. Such as, housing given by local councils. |
| LA Mental Health Support | Mental health support given from local authorities. Such as, community mental health teams. |
| 3rd Sector Drug and Alcohol Services | Drug and alcohol support given by third sector/ charities. Such as, NEWID drug and alcohol support and BAROD. |
| Statutory Education | Education experiences such as TAITH (International exchange programme). |
| Employer Support | Employee wellbeing services, such as the People Health and Wellbeing Service by Cardiff and Vale Health Board for their employees. |
| SARC | Sexual Assault Referral Centres. Supporting those who have experienced sexual assault. |
| SPOA | Single point of access. An NHS system where there is a single point of contact where patients can have a clinical assessment by a multidisciplinary team. Patients can be referred on or cases can be held. |
| 3rd Sector Anonymous Crime Reporting | Online anonymous crime reporting led by Crime Stoppers. CYP service called Fearless. |
| 3rd Sector Mental Health Support | Mental Health support led by third sector organisations such as, MIND, the Amber Project, The Samaritans, Concern Cymru and PLATFFORM. |
| Statutory Youth Support | Support from statutory bodies and local authorities such as, Councils Children’s Care Services and Youth services like Cardiff and Vale Early Help Services and Cardiff and Vale Young Wellbeing. |
| Citizens Advice | A UK-based organisation aiding people with legal, housing, debt and other services. |
| NHS Mental Health Support | Mental Health support given by NHS organisations, such as Children and Adolescent Mental Health Services (CAMHS) and Adult Mental Health Support Services and Mental Health Liaison Nurses. |
| NHS Primary | Primary care, e.g. GPs, pharmacies, dentists and opticians. |
| LA housing support | Housing support given by local authorises such as, houses given by councils, tenancy support units and LA/council-based homelessness support. |
| GP | General practitioner. |
| Statutory Youth Services | Local Government support for young people. |
| Local Authority Environmental Heath | Public health inspectors who ensure health and safety is upheld in the workplace. |
| Probation | Probation Services supporting those who are on probation. |
| School | Letting schools know about support or support from school nurses. |
| Statutory Family Support | LA/council-based family services. |
| CVPT | Cardiff Violence Prevention Team. They can refer onto the focused agencies, or they can hold cases. |
| Police | Police reporting with consent, police support, police services and in cases where a weapon is used non-consensual police reporting. |
| Social Worker | Individuals that can provide support and safeguarding to vulnerable people. |
| VPT Supported | Support given to patients and cases held. |
| Safeguarding | Statutory/LA safeguarding, such as council-based safeguarding for example, educational safeguarding. |
| Swansea VPT | Swansea Violence Prevention Team. They can refer onto the focused agencies, or they can hold cases. |
| Multi-agency Referrals | Multi-agency referrals and forms, such as, referral into a multi-agency risk assessment conference (MARAC). |
| 3^rd^ Sector Victim Support | 3^rd^ sector support for violence-related attendances including domestic violence services. Such as, Victim Focus/Support, New Pathways and St Giles. |
| NHS Drugs and Alcohol | Drug and alcohol services led by NHS. Such as, Cardiff and Vale Drugs and Alcohol Services (CAVDAS). |
| 3^rd^ Sector Drug and Support | 3^rd^ sector support and treatment for drug-related issues. |
| 3^rd^ Sector Youth Support | 3^rd^ sector support for children and young people. Such as, Media Academy Cymru and Action for Children. |
| Statutory Victim Support | Support for victims led by LA/statutory bodies. Such as, council-based support for domestic violence. |
| Advocate | Independent domestic violence advocates (IDVAs) and independent sexual violence advocates (ISVAs). |
| Police Report | Consensual police reporting or non-consensual police reporting if a weapon was used. |
| NHS Secondary | Hospital and community care. Such as, outpatient services. |
| NHS Homeless Support | NHS based support for homelessness. Such as, the Cardiff and Vale Homeless Nursing Team. |
| Statutory Adult Services | LA/statutory support for adults. |
| NHS Youth Support | NHS based support for children and young people. Such as, Cardiff and Vale UHB Early Help Services and young people’s/paediatric IDVAs. |
| 3^rd^ Sector Gender and Sexuality Support | Gender and sexuality support led by 3rd sector organisations. Such as, Umbrella Cymru’s Gender and Sexual Diversity Support Specialists. |
| 3^rd^ Sector Refugee Support | Support for refugees and asylum seekers. Such as, Oasis. |
| Social Prescribing | Support via community activities based on what the patient enjoys. Such as, boxing and football clubs or gaming. |
| CMET | Contextual, Missing, Exploited and Trafficked. A multi-agency panel for safeguarding. |
| IRF | Inpatient Referral Facility, Facilities with acute care services. May be for drugs and alcohol or mental health. |

# Supplement 3: Study Data Sets

The Secure Anonymised Information Linkage (SAIL) Databank contains health, social and education data on over three million residents of Wales. ^3 4^ Information governance for SAIL is overseen by an independent Information Governance Review Panel (IGRP). Core data sets can be accessed following IGRP approval; for access to restricted core datasets, permission from the data providers is required in addition to IGRP approval. Robust policies, structures, controls and special software are in place to protect privacy through a reliable matching, anonymisation and encryption process achieved in conjunction with the National Health Service Wales Informatics Service (NWIS) using a split file approach. ^5^ For each data set within the SAIL Databank, each included individual is assigned an Anonymised Linking Field (ALF) that enables cross-linking. The ALF is based on an individual’s National Health Service (NHS) number or a combination of unique identifiers such as name, gender, and date of birth. The smallest geographical area for which data are already linked and may be released from the SAIL Databank, after disclosure control to take account such as small numbers, is the Lower Layer Super Output Area (LSOA). LSOA codes can be used to link to reference data such as deprivation scores, Welsh Index of Multiple Deprivation (WIMD 2019) based on the LSOAs of the 2011 Census and Office for National Statistics (ONS) settlement types such as village, town, and urban.

## Data Tables

Table S2 – Description of the data tables individually linked for the evaluation

| Emergency Department Data Set | EDDS | Attendance and clinical information for all Emergency Department attendances.  https://healthdatagateway.org/en/dataset/299 |
| --- | --- | --- |
| Welsh Longitudinal General Practitioner Data Set | WLGP | Attendance and clinical information for all general practice interactions: including patient symptoms, investigations, diagnoses, prescribed medication and referrals to tertiary care.  https://healthdatagateway.org/en/dataset/355 |
| Patient Episode Database Wales | PEDW | The database contains all inpatient and day case activity undertaken in NHS Wales plus data on Welsh residents treated in English Trusts.  https://healthdatagateway.org/en/dataset/318 |
| Welsh Demographic Service | WDS | Register of all individuals registered with a Welsh GP, includes individuals anonymised address and practice history.  https://healthdatagateway.org/en/dataset/359 |
| Census 2021 | CEN21 | A census in the UK is a count of all people and households. This census in the UK was held on 21 March 2021.  https://healthdatagateway.org/en/dataset/361 |
| Census 2011 | CEN11 | A census in the UK is a count of all people and households. This census in the UK was held on 27 March 2011.  https://healthdatagateway.org/en/dataset/335 |
| Welsh Index of Multiple Deprivation Quintiles | WIMD | WIMD is the Welsh Government’s official measure of relative deprivation for small areas in Wales, based eight domains including income, employment, health, and access to services. Typically grouped into fifths or “quintiles”, the WIMD is included in several NHS datasets. |

## Derived Variables

Table S3 – Derived variables

| Characteristic | Source |
| --- | --- |
| Age (from Week of Birth, WoB) | WDS |
| Sex  • Male  • Female | WDS |
| Ethnicity  • Asian (Bangladeshi, Chinese, Indian, Pakistani, Other Asian)  • Black (Caribbean, African, Other Black)  • Mixed (White and Asian, White and Black African, White and Black Caribbean, Other Mixed or Multiple ethnic groups)  • White (English, Welsh, Scottish, Northern Irish, British, Irish, Gypsy or Irish Traveller, Roma, Other White)  • Other (Arab, Any other ethnic group)  Ethnicity was derived from the 2021 Census first, if missing then the 2011 Census, and if still missing then from, in order, PEDW, WLGP, EDDS. | 2011, 2021 Census, WLGP, PEDW, EDDS |
| Quintile of Residential Deprivation | WIMD |
| Urban/rural residential classification | WDS |
| Discharge from ED  • Discharge home  • Admitted into hospital | EDDS |
| Previous month ED attendances | EDDS |
| Time to inclusion. The date of attendance for each patient included in analyses varied after the cohort start date (1 November 2019). Date of inclusion was derived to enable a match between treatment arms and those attending at similar times. | EDDS |

# Supplement 4: Matching

The purpose of matching control and intervention patients is to allow derivation of the average treatment effect under the assumption that allocation is not conditional on observed confounders. Matching on values of covariates between the treated and untreated groups avoids the bias introduced by covariates that influence the outcome variable. The goal being to find a subset of data that is closest to an exact match on observed covariates. ^6 7^ Given the likely contribution of intersectional risk factors, the primary analysis was conducted using Coarsened Exact Matching (CEM), with a one-to-many (1:M) ratio where M is all available controls, with secondary analyses undertaken restricting CEM to 1:1, and Propensity Score Matching in sensitivity analyses.

In CEM, the coarsening represents the process of reducing continuous variables, such as age, to discrete categories that are then used to match across treatment groups. An attempt then made to find exact matches across coarsened, categorical and binary variables. Our rational for this approach being that, for example, being young, male, residing in a deprived neighbourhood is uniquely different to being young, male and residing in an affluent neighbourhood. In addition, CEM typically achieves superior covariate balance compared to alternative methods, including propensity score matching. There are, furthermore, theoretical advantages to CEM: explicitly bounding model dependence and causal effect estimation error through ex ante user choice, computational efficiency even with very large datasets, and invariance to measurement error.

However, CEM’s most critical disadvantage is substantial data loss, particularly in high-dimensional settings. This aggressive pruning leads to high bias and low precision of effect estimates due to sparse data bias, with CEM producing the least precise estimates among compared methods and severely misidentifying average treatment effects. The method performs poorly with large databases containing rich covariate information, in high-dimensional datasets where it finds exponentially fewer matches, and when common support is poor. Evidence suggests CEM should not be used as the sole balancing method but may serve effectively as a sensitivity analysis or in combination with other approaches when exact balance on a limited set of discrete confounders is scientifically critical. For categorial covariates, exact matching is a sensible alternative. Here a match is considered acceptable only if the values of all covariates are equal between treated and untreated potential matches. Continuous covariates were coarsened into categorial covariates. For matching, several covariate categories were further grouped to form larger (and fewer) categories, improving efficiency. ^6-8^ This approximates to a fully blocked experiment.

Propensity score matching reduces the multi-dimensional space of covariates to a single summary covariate, the propensity score, typically derived using a logistic equation and performing regression on the treatment group for the covariates chosen to match on. The proximity of a potential match to the given subject is estimated in terms of the closeness of their propensity scores. This method simplifies subsequent analyses as matching refers only to one variate. However, propensity score matching can increase, rather than decrease, the imbalance of the covariates in the samples. ^6^ A challenge is optimising the trade-off between the quality of matches and the sample size. A larger sample reduces sampling error and can increase the study power, but including matches of lower quality may lead to greater residual imbalance. ^8^

## Coarsening

The Stata V18 implementation of CEM ^9^ was used to coarsen and match by allocation. Coarsening and matching was repeated for each of the primary, secondary and sensitivity analyses. However, there were two adjustments pre-coarsening in response to low numbers. Five or more prior month ED attendances were grouped into a single 5+ group and patients 65 years and older were group into a single 65 years of age and older group.

## Variables included in Coarsened Exact Matching

- Age in years
  - CEM coarsening across the whole age range used up to 64.9 years, with subsequent categories combined from 65 and over years of age, due to low number in these latter categories.
- Sex
  - Male
  - Female
- Ethnicity, Office for National Statistics five categories
  - Asian: Asian British or Asian Welsh, Bangladeshi
  - Black: Black British, Black Welsh, Caribbean or African, African
  - Mixed: Multiple ethnic groups, White and Asian
  - White: English, Welsh, Scottish, Northern Irish or British
  - Other: Any other ethnic group
- Residence
  - Urban
  - Rural
- Welsh Index of Multiple Deprivation
  - 1 Least Deprived
  - 2
  - 3
  - 4
  - 5 Most Deprived
- Time to inclusion
  - Days, denoting the difference between the date of index assault-related attendance necessary for inclusion in the study cohort and study start date
- Admitted to hospital
  - Admitted to same hospital within LHB OR Admitted to other hospital within LHB OR patient dead on arrival
  - Everything else (incl. transferred to hospital in different LHB, referred to outpatient department, referred to GP, referred to other healthcare professional, no planned follow-up, planned follow-up at A&E, patient self-discharge without clinical consent, died in department)
- High Intensity User
  - Count of ED attendances in the preceding month up to index assault-related attendance and inclusion in the study, >=5 were combined into one category due to low numbers.

CEM means that all continuous variables are coarsened into categorical variables. The number of cuts are per Sturges rule. ^10^

# Supplement 5: Time Series and Discontinuous Risk

Accounting for periods when individuals are not at risk is an essential consideration in repeated time-to-event models. ^11 12^ The clearest example in the current context is ensuring time at risk does not extend beyond date of death or originates before birth. Similarly, time at risk will also be right side censored if they move away from Wales, as only residents of Wales are eligible for inclusion. With no adjustment for these discontinuous risk intervals, the time at risk will be incorrect, increasing a greater likelihood of Type II errors. How patients are routed through emergency care pathways in Wales also influences time at risk. ED attendances are mainly determined by the acuity of the patient’s condition and the urgency with which they need to be seen. These decisions can be made by the Welsh Ambulance Service Trust (WAST), a Minor Injuries Unit (MIU) or in the local ED. It is feasible that a patient initially attends a local ED to be stabilised, is assessed, and requires referral to a Major Trauma Centre (MTC), or Trauma Unit (TU). Each MTC and TU are attached to an ED, and therefore in response to severe injury, patients are registered in more than one ED if they are referred from an ED without trauma facilities, to EDs that are attached to a TU or MTC. In Wales, emergency care in Wales is provided in MIUs, EDs (Local Emergency Hospitals, LEH, and Rural Trauma Facilities, RTFs), TUs, and MTCs. There is one MTC in Cardiff UHW, which services South and West Wales, and South Powys, and acts as a TU for the local population. Morriston Hospital in Swansea is a TU, but with additional specialist services (e.g., orthoplastics) meaning some patients otherwise destined for the MTC would be referred there instead. North Wales is serviced by the MTC in the Royal Stoke University Hospital in England (any attendance by a Welsh resident will also be included in the Welsh ED data). Patient admission is described using spells and super-spells. A spell represents patient care by specialty, and a super-spell is a collection of spells – two spells are included in the same super-spell if admission to the second specialty is within 48 hours of discharge from the first. ED attendances included within the same super-spell (e.g., transfer from an ED to an MTC) will therefore be attributable to the same initiating event, whether planned or unplanned. Therefore, only unscheduled attendances at the initiation of a super-spell are included, and additional ED attendances within the same super-spell will be analytically censored, as they are a continuation of the initial attendance and not therefore independent. The duration of a super-spell therefore constitutes a discontinuity in a patient’s exposure to risk, which is then accommodated in our analytic approach.

# Supplement 6: Data Derivation

## Data flow diagram for Coarsened Exact Matching (CEM)

Data sources – an uploaded dataset containing intervention participants identified by the violence prevention team (VPT), WDSD, EDDS, PEDW.

The following contains the tables relating to matching for the three primary study populations.

CEM means that all continuous variables are coarsened into categorical variables. The number of cuts is per Sturges rule.

The following variables were included in the CEM

- Age (CEM coarsening across the whole age range used up to 64.9 years and then 65+ years grouped together as a population of interest)
- Sex
- Ethnicity (white/Asian/other vs mixed/black)
- Urban rural (urban vs rural)
- WIMD quintiles
- Time to inclusion
- Admitted to hospital
- ED usage in the month before inclusion in the study. $x\geq5$ were grouped into a single category.

There are 14,604 control participants, no exclusions are made to these participants pre-matching.

### Intention To Treat

Table S4 – ITT CEM.

| Participants matched during CEM | Control participants, n (%) | | Intervention participants, n (%) | |
| --- | --- | --- | --- | --- |
| Matched | 8,927 | (35.2%) | 5,965 | (83.0%) |
| Unmatched | 5,677 | (64.8%) | 1,226 | (17.0%) |
| Total | 14,604 |  | 7,191 |  |

### Engaged with the Violence Prevention Team (VPT) – PP group 3

Table S5 – Excluding participants who received a dosage of the intervention that was not engaging with the VPT.

| Intervention participants included in matching | Participants, n (%) | |
| --- | --- | --- |
| Intervention – engaged with the VPT (included) | 2,539 | (35.3%) |
| Intervention – did not engage with the VPT (excluded) | 4,652 | (64.7%) |
| Total | 7,191 |  |

Table S6 – Engaged with the VPT CEM.

| Participants matched during CEM | Control participants, n (%) | | Intervention participants, n (%) | |
| --- | --- | --- | --- | --- |
| Matched | 6,196 | (42.4%) | 2,069 | (81.5%) |
| Unmatched | 8,408 | (57.6%) | 470 | (18.5%) |
| Total | 14,604 |  | 2,539 |  |

### Referred out by VPT – PP group 4

Table S7 – Excluding participants who received a dosage of the intervention that was not being referred out by VPT.

| Intervention participants included in matching | Participants, n (%) | |
| --- | --- | --- |
| Intervention – referred out by VPT (included) | 1,506 | (20.9%) |
| Intervention – not referred out by VPT (excluded) | 5,685 | (79.1%) |
| Total | 7,191 |  |

Table S8 – Referred out by VPT CEM.

| Participants matched during CEM | Control participants, n (%) | | Intervention participants, n (%) | |
| --- | --- | --- | --- | --- |
| Matched | 5,142 | (35.2%) | 1,227 | (81.5%) |
| Unmatched | 9,462 | (64.8%) | 279 | (18.5%) |
| Total | 14,604 |  | 1,506 |  |

## Outcome Data

### Step 1 – EDDS Data

Data sources – an uploaded dataset containing intervention participants identified by the violence prevention team (VPT), WDSD, EDDS, PEDW.

The following file contains a step-by-step breakdown of the derivation of the outcome dataset for effectiveness and cost-effectiveness. ALF_PE is the variable name for the anonymised linkage field (ALF), referred to elsewhere.

Table S9 – ED attendances with a non-missing ALF_PE (from EDDS_EDDS).

| ED attendances with an ALF_PE | Records, n (%) | | Unique ALF_PE, n | |
| --- | --- | --- | --- | --- |
| Yes | 14,475,165 | (96.2%) | 3,310,370 |  |
| No | 565,806 | (3.8%) | n/a |  |
| Total | 15,040,971 |  | 3,310,370 |  |

ECLIPSE does not consider a missing ALF_PE as a unique ALF_PE and therefore it is n/a. In SPSS, this would be considered as a unique ALF_PE.

Table S10 – ED attendances on or after 01/11/2019.

| ED attendances with administrative arrival date >= 01/11/2019 | Records, n (%) | | Unique ALF_PE, n | |
| --- | --- | --- | --- | --- |
| Yes | 4,527,428 | (31.3%) | 1,802,935 |  |
| No | 9,947,737 | (68.7%) | 2,783,890 |  |
| Total | 14,475,165 |  | 3,310,370 |  |

Unique ALF_PE may not add up to the total because ALF_PEs are unique in both datasets.

Table S11 – ED attendances classified as new attendances.

| ED attendances classified as | Records, n (%) | | Unique ALF_PE, n | |
| --- | --- | --- | --- | --- |
| New | 4,384,528 | (96.8%) | 1,801,950 |  |
| Follow-up or pre-operative assessment | 142,900 | (3.2%) | 99,917 |  |
| Total | 4,527,428 |  | 1,802,935 |  |

Unique ALF_PE may not add up to the total because ALF_PEs are unique in both datasets.

We identified a cohort of 21,795 participants (ALF_PE) meeting the inclusion criteria to link to. This cohort was derived prior to the outcome file.

Table S12 – ED attendances linked to a study participant.

| ED attendances linked to a study participant | Records, n (%) | | Unique ALF_PE, n | |
| --- | --- | --- | --- | --- |
| Linked | 114,687 | (2.6%) | 21,722 |  |
| Not linked | 4,269,841 | (97.4%) | 1,779,858 |  |
| Total | 4,384,528 |  | 1,801,950 |  |

There are 73 less participants identified than in the cohort (21,795). As we will have linked to all ED records, the missing 73 participants do not have any records in EDDS. Therefore, these participants were identified via the uploaded VPT dataset. We will now identify how many participants do not have an ED attendance associated with their date of inclusion, initial assault related attendance (ARA).

Table S13 – ED attendances linked to the participant’s date of inclusion.

| ED attendances linked to date of inclusion | Records, n (%) | | Unique ALF_PE, n | |
| --- | --- | --- | --- | --- |
| Linked | 23,836 | (2.1%) | 21,392 |  |
| Not linked | 90,851 | (97.9%) | 17,693 |  |
| Total | 114,687 |  | 21,722 |  |

Unique ALF_PE may not add up to the total because ALF_PEs are unique in both datasets.

There are 403 participants who do not have an ED attendance associated with their date of inclusion; an additional 330 participants to identified in table 4. We identified the 403 participants missing a record signifying their date of inclusion a generated a record. The total number of records will be (114,687 + 403 =) 115,090 and participants (21,722 + 73 =) 21,795.

Table S14 – ED attendances in the participant’s study period.

| ED attendances >= date of inclusion | Records, n (%) | | Unique ALF_PE, n | |
| --- | --- | --- | --- | --- |
| Yes | 74,197 | (64.5%) | 21,795 |  |
| No | 40,893 | (35.5%) | 12,396 |  |
| Total | 115,090 |  | 21,795 |  |

Unique ALF_PE may not add up to the total because ALF_PEs are unique in both datasets.

#### Hospital Admissions

To identify hospital admissions, we used the dataset PEDW_SINGLE_DIAG. The implications of using this dataset are that this excludes hospital admissions where there were no diagnoses recorded during the admissions. Whilst this scenario is unlikely, we have identified scenarios where this happens. When reviewing these records, we identified that there are no HRG codes attributed to them, costing variables required for the cost-effectiveness analysis. Therefore, costings could only be attributed per a day/overnight perspective. We concluded that it was sufficient to use the PEDW_SINGLE_DIAG dataset with the identified limitations briefly mentioned above.

Table S15 – Hospital admissions with a non-missing ALF_PE (from PEDW_SINGLE_DIAG).

| Hospital admissions with an ALF_PE | Records, n (%) | | Unique ALF_PE, n | |
| --- | --- | --- | --- | --- |
| Yes | 111,075,538 | (97.7%) | 3,745,187 |  |
| No | 2,629,836 | (2.3%) | n/a |  |
| Total | 113,705374 |  | 3,745,187 |  |

ECLIPSE does not consider a missing ALF_PE as a unique ALF_PE and therefore it is n/a. In SPSS, this would be considered as a unique ALF_PE.

Table S16 – Hospital admissions on or after 01/11/2019 but a maximum year of 2024.

| Hospital admissions | Records, n (%) | | Unique ALF_PE, n | |
| --- | --- | --- | --- | --- |
| Start date >= 01/11/2019 & year of start date <= 2024 | 25,399,215 | (22.9%) | 1,282,917 |  |
| Start date < 01/11/2019 | 85,676,039 | (77.1%) | 3,438,477 |  |
| Year of start date > 2024 | 147 | (<0.1%) | 44 |  |
| Start date is missing | 137 | (<0.1%) | 42 |  |
| Total | 111,075,538 |  | 3,745,187 |  |

Unique ALF_PE may not add up to the total because ALF_PEs are unique in both datasets.

Table S17 – Unique hospital admissions.

| Unique hospital admissions | Records, n (%) | | Unique ALF_PE, n | |
| --- | --- | --- | --- | --- |
| Yes | 3,831,109 | (15.1%) | 1,282,917 |  |
| No | 21,568,106 | (84.9%) | 1,136,341 |  |
| Total | 25,399,215 |  | 1,282,917 |  |

Unique ALF_PE may not add up to the total as a unique person can be in both datasets.

Table S18 – Hospital admission linked to a study participant.

| Hospital admissions linked to a study participant | Records, n (%) | | Unique ALF_PE, n | |
| --- | --- | --- | --- | --- |
| Linked | 29,977 | (0.8%) | 10,005 |  |
| Not linked | 3,801,132 | (99.2%) | 1,272,912 |  |
| Total | 3,831,109 |  | 1,282,917 |  |

Table S19 – Hospital admission in the participant’s study period.

| Hospital admissions with a start date >= date of inclusion | Records, n (%) | | Unique ALF_PE, n | |
| --- | --- | --- | --- | --- |
| Yes | 17,193 | (57.4%) | 6,901 |  |
| No | 12,784 | (42.6%) | 5,358 |  |
| Total | 29,977 |  | 10,005 |  |

Unique ALF_PE may not add up to the total as a unique person can be in both datasets.

### Step 2 – EDDS and Hospital Admissions

Table S20 – ED attendances within a hospital admission.

| ED attendances not within a hospital admission | Records, n (%) | | Unique ALF_PE, n | |
| --- | --- | --- | --- | --- |
| Yes | 74,101 | (99.9%) | 21,795 |  |
| No | 96 | (0.1%) | 52 |  |
| Total | 74,197 |  | 21,795 |  |

Unique ALF_PE may not add up to the total because ALF_PEs are unique in both datasets.

Table S21 – ED attendances linked to a hospital admission.

| ED attendance linked to a hospital admission | Records, n (%) | | Unique ALF_PE, n | |
| --- | --- | --- | --- | --- |
| Linked | 6,045 | (8.2%) | 3,721 |  |
| Not linked | 68,056 | (91.8%) | 21,426 |  |
| Total | 74,101 |  | 22,198 |  |

Unique ALF_PE may not add up to the total because ALF_PEs are unique in both datasets.

Table S22 – Hospital admissions linked to an ED attendance.

| Hospital admissions linked to an ED attendance. | Records, n (%) | | Unique ALF_PE, n | |
| --- | --- | --- | --- | --- |
| Linked | 5,697 | (33.1%) | 3,721 |  |
| Not linked | 11,496 | (66.9%) | 4,896 |  |
| Total | 17,193 |  | 6,901 |  |

Unique ALF_PE may not add up to the total because ALF_PEs are unique in both datasets.

There is a difference of 348 records linked between the two. This is because there are 348 instances where an hospital admission is linked more than once to an ED attendance. This is because ED attendances have been linked by 2 methods.

1. ED attendances are linked to a hospital admission if they end on the same date as the hospital admission starts.
2. ED attendances finished after 22:00 can also been linked to a hospital admission if the hospital admission starts the day after the ED attendance ended. The rational being to allow a window for people to make their way from ED to hospital.

No date is recorded in PEDW and therefore it was not possible to apply a time restriction to the hospital admissions.

### Step 3 – Unique Attendances

Table S23 – Unique ED attendances by date and time.

| Unique ED attendances by date and time | Records, n (%) | | Unique ALF_PE, n | |
| --- | --- | --- | --- | --- |
| Unique | 70,787 | (95.5%) | 21,795 |  |
| Duplicate | 3,314 | (4.5%) | 2,810 |  |
| Total | 74,101 |  | 21,795 |  |

Unique ALF_PE may not add up to the total because ALF_PEs are unique in both datasets.

Table S24 – Unique ED attendance by date.

| ED attendance by date | Records, n (%) | | Unique ALF_PE, n | |
| --- | --- | --- | --- | --- |
| Unique | 69,382 | (98.0%) | 21,795 |  |
| Duplicate | 1,405 | (2.0%) | 1,259 |  |
| Total | 70,787 |  | 21,795 |  |

Unique ALF_PE may not add up to the total because ALF_PEs are unique in both datasets.

Due to low counts and risk of inadvertent disclosure, the following steps have been condensed into a single stage.

ED attendances with at risk start day >= 0 were excluded in 2 steps:

- ED attendance A’s starts and ends within ED attendance B’s start and end dates.
- ED attendance A’s starts within ED attendance B’s start and end dates. The end date at this stage is derived as the maximum end date of the ED attendance or hospital admission (if linked). As ED attendance starts within ED attendance B, the days at risk are negative because the attendance starts before the previous one ends.

These steps both investigate negative days at risk however we first excluded ED attendances within another ED attendance before considering hospital admissions linked to the ED attendances.

Table S25 – ED attendances within a at risk start days >= 0.

| ED attendance not within another ED attendance | Records, n (%) | | Unique ALF_PE, n | |
| --- | --- | --- | --- | --- |
| Yes | 69,358 | (>99.9%) | 21,795 |  |
| No | 24 | (<0.1%) |  |  |
| Total | 69,382 |  | 21,795 |  |

During these steps, we ensured hospital admissions were only linked to a single ED attendance too. The hospital admissions are excluded in the steps described above, as well as excluding duplicate hospital admissions. They have been grouped as such to not prevent a disclosure risk.

Table S26 – Hospital admissions included following identifying unique ED attendances.

| Hospital admissions included in the dataset moving forwards | Records, n (%) | | Unique ALF_PE, n | |
| --- | --- | --- | --- | --- |
| Included | 5,662 | (93.7%) | 3,707 |  |
| Excluded | 383 | (6.3%) |  |  |
| Total | 6,045 |  | 3,721 |  |

Unique ALF_PE may not add up to the total because ALF_PEs are unique in both datasets.

### Step 4 – WLGP and Residency

Table S27 – Welsh GP residency records (from WDSD_PER_RESIDENCE_GPREG).

| Welsh GP residency | Records, n (%) | | Unique ALF_PE, n | |
| --- | --- | --- | --- | --- |
| With an ALF_PE | 26,551,107 | (96.2%) | 5,876,890 |  |
| Without an ALF_PE | 36,978 | (3.8%) | n/a |  |
| Total | 26,588,085 |  | 5,876,890 |  |

ECLIPSE does not consider a missing ALF_PE as a unique ALF_PE and therefore it is n/a. In SPSS, this would be considered as a unique ALF_PE.

We will refer to “GP residency” as “residency” for simplicity moving forward.

Table S28 – Welsh residency starts before or on 31/07/2024.

| Welsh residency start date <= 31/07/2024 | Records, n (%) | | Unique ALF_PE, n | |
| --- | --- | --- | --- | --- |
| Yes | 26,510,504 | (99.8%) | 5,874,182 |  |
| No | 40,603 | (0.2%) | 38,310 |  |
| Total | 26,551,107 |  | 5,876,890 |  |

Unique ALF_PE may not add up to the total because ALF_PEs are unique in both datasets.

Table S29 – Welsh residency ended after or on 01/11/2019 or ongoing.

| Welsh residency and date >= 01/11/2019 or missing (residency ongoing) | Records, n (%) | | Unique ALF_PE, n | |
| --- | --- | --- | --- | --- |
| Yes | 6,365,005 | (24.0%) | 3,772,336 |  |
| No | 20,145,499 | (76.0%) | 5,357,948 |  |
| Total | 26,510,504 |  | 5,876,890 |  |

Unique ALF_PE may not add up to the total because ALF_PEs are unique in both datasets.

Table S30 – Welsh residency has a Welsh LSOA code.

| Welsh residency has a Welsh LSOA code | Records, n (%) | | Unique ALF_PE, n | |
| --- | --- | --- | --- | --- |
| Yes | 5,682,165 |  | 3,745,962 |  |
| No | 682,840 |  | 542,376 |  |
| Total | 6,365,005 |  | 3,772,336 |  |

Unique ALF_PE may not add up to the total because ALF_PEs are unique in both datasets.

The Welsh residency records are then collapsed into a single residency record for each ALF_PE (3,745,962 records). In this collapse, the start date is derived as the minimum start date and the end date is derived as the maximum end date across all their records. This may mean that there are gaps in a participants residency, where they are no resident in Wales, however the collapse will consider them as a single Welsh resident.

### Step 5 – At Risk Period

Table S31 – ED attendances within the participant’s study period.

| ED attendance within the participant’s study period | Records, n (%) | | Unique ALF_PE, n | |
| --- | --- | --- | --- | --- |
| Yes | 69,175 | (99.7%) | 21,795 |  |
| No | 183 | (0.3%) | 80 |  |
| Total | 69,358 |  | 21,795 |  |

Unique ALF_PE may not add up to the total because ALF_PEs are unique in both datasets.

To model the final at-risk period, time after final ED attendance until censoring, we add a record (+21,795) for all participants and then identify who does not need a final at-risk period record based off the days at-risk from previous record.

Table S32 – Final at-risk period of interest.

| Final at-risk period’s days at-risk > 0 | Records, n (%) | | Unique ALF_PE, n | |
| --- | --- | --- | --- | --- |
| Yes | 21,633 | (99.3%) | 21,633 |  |
| No | 162 | (0.7%) | 162 |  |
| Total | 21,795 |  | 21,795 |  |

If the final at-risk period has a days at-risk < 0 then it starts within the previous record. Therefore, like before need to be excluded. If the final at-risk period has a days at-risk = 0 then the previous record was either the cause of censoring or was happening and they were censored during the record. Therefore, we have a total of (69,175 + 21,633 =) 90,808 records in the final outcome dataset before matching.

# Supplement 7: Patient and Public Involvement

Public, Patient Involvement (PPI) is reported according to GRIPP2 guidance. ^13^

**PPI Aims**

PPI was undertaken to aid project development, prosecution, interpretation and dissemination.

**PPI Methods**

PPI groups included one group that had precious experience of PPI work; this group was recruited from Service Users for Primary and Emergency Care Research (SUPER). SUPER supported and provided feedback to the research team during project development and on the research team’s plans to include those with lived and living experience who had not previously contributed to research.

As Violence Prevention Teams (VPTs) often support those who may have underlying psychosocial vulnerabilities and for whom VPT engagement may be their first clinical contact we sought to include PPI members with lived or living experience. They were recruited from third sector organisations that support vulnerable and hard to reach groups. One organisation provides support to those with lived or living experience of homelessness (The Wallich) and one provides support to those with lived experience of domestic and sexual violence (Welsh Woman’s Aid). Ethnicity was often discussed by PPI members; therefore, a further PPI session was conducted with a third sector organisation supporting those from black and ethnic minority groups who have experienced violence (BAWSO).

PPI was undertaken by two researchers from the research team with support from two PPI co-investigators. Two sessions were conducted with SUPER and two sessions were conducted each with The Wallich and Welsh Woman’s Aid. One follow-up session was undertaken with BAWSO. Participants were initially provided with information about the study in plain English. SUPER’s session was based on the original proposal, prior to submission, and they additionally advised on the suitability of PPI materials for those who had not taken part in PPI before.

**PPI Results**

SUPER

SUPER contributed to the initial development of the research proposal, and the development of the research protocol, in the following ways:

- Initial consultation in planning the funding application.
  - The research proposal was reviewed in July 2021, and the input and advice contributed to a successful funding application. The research team also responded to the formative comments when developing the protocol and continued to build on them.
- Development and refinement of the protocol
  - Adapt the protocol to clarify the type of violence included in this study.
  - Consider the experiences of different ethnic groups and violence.
  - Consider the reasons why patients may not disclose violence.
  - PPI involvement in future diffusion and dissemination activity would lend credence to the project’s communication strategy.

**3^rd^ Sector PPI (Welsh Woman’s Aid and The Wallich)**

- Data on ethnicity should be included so that racial violence could be considered.
- To consider patient ascertainment and therefore eligibility for the intervention in ED, as some victims may not realise that they are victims of violence. From this feedback we planned an ascertainment study looking at whether HVIPs improve ascertainment.
- Some patients might choose to avoid the police and therefore be less reluctant to receive support from the police, or police aligned services. We might explore this in any data available.
- That there are unique challenges for those who are disabled, both in terms of ability to engage and the nature of the support required.
- Some patients, notably those with children, may be less willing to engage as they would not want to risk losing their home or children.

**3^rd^ Sector PPI (BAWSO)**

- Cultural differences are important to consider. In terms of violence, some are told to put up with it and that calling the police would dishonour the family. Divorce is also sometimes not an option.
- Potential barriers include fear of deportation, fear of people in the home country and fear children will be taken away. All are barriers to disclosure which may affect the VPTs.
- Language barriers may affect disclosure. The perpetrator may act as a translator to influence control.
- Fear of police in communities, specifically those who have the potential to be deported. This can also lead to fear of the health service due to their association with the police.
- Other barriers of health care may be due to experiences in their countries. Some may have to pay for healthcare and if they cannot afford it police would get involved.

**Discussion and Conclusions**

## Reflections

- Adequate funding to pay public involvement members for their time was available, which provided incentive for PPI group members to take part.
- Sessions were held as hybrid events, enabling access for those who could not travel.
- There were existing relationships between the research team and third sector organisations which made initial contact easier.
- The third sector organisations had their own networks, who were experienced in consultations and service improvement. This made it easier to recruit public members to be involved in our research. This also meant the members of the PPI groups were vocal and skilled at communicating their opinions on the research and the violence prevention teams.

There were some limitations with PPI sessions:

- People with lived experience were not involved in developing the Violence Prevention Team service, so the PPI groups discussed how they would have developed the service differently if they had been able to have input. However, the research team are not in the position to be able to change this service.

## Conclusion

PPI members have provided the research team with information on the experiences of NHS patients with psychosocial vulnerabilities, enabling an understanding of barriers to disclosure and how patients with underlying psychosocial vulnerabilities would like support.

PPI members suggested that patient disclosure may be an issue with the VPTs effectiveness, thus, we looked at whether the VPTs would increase the likelihood and whether any specific groups would be more likely to disclose to the VPTs, compared to under usual care. This is subject to an additional research output.

PPI suggested the consideration of ethnicity; therefore, we included administrative data sets in the study such as the two Census datasets.

Valuable insights from PPI about dissemination from which a dissemination event with stakeholders and PPI members was planned, and a lay briefing document sharing a plain English summary.

# Supplement 8: RECORD and STROBE checklist

The RECORD statement – checklist of items, extended from the STROBE statement, that should be reported in observational studies using routinely collected health data. ^14^

|  | Item No. | STROBE items | Location in manuscript where items are reported | RECORD items | Location in manuscript where items are reported |
| --- | --- | --- | --- | --- | --- |
| Title and abstract | | | | | |
|  | 1 | (a) Indicate the study’s design with a commonly used term in the title or the abstract (b) Provide in the abstract an informative and balanced summary of what was done and what was found |  | RECORD 1.1: The type of data used should be specified in the title or abstract. When possible, the name of the databases used should be included.  RECORD 1.2: If applicable, the geographic region and timeframe within which the study took place should be reported in the title or abstract.  RECORD 1.3: If linkage between databases was conducted for the study, this should be clearly stated in the title or abstract. | Title and Abstract |
| Introduction | | | | | |
| Background rationale | 2 | Explain the scientific background and rationale for the investigation being reported |  |  | Introduction |
| Objectives | 3 | State specific objectives, including any prespecified hypotheses |  |  | Introduction |
| Methods | | | | | |
| Study Design | 4 | Present key elements of study design early in the paper |  |  | Methods |
| Setting | 5 | Describe the setting, locations, and relevant dates, including periods of recruitment, exposure, follow-up, and data collection |  |  | Methods |
| Participants | 6 | *(a) Cohort study* - Give the eligibility criteria, and the sources and methods of selection of participants. Describe methods of follow-up  *Case-control study* - Give the eligibility criteria, and the sources and methods of case ascertainment and control selection. Give the rationale for the choice of cases and controls  *Cross-sectional study* - Give the eligibility criteria, and the sources and methods of selection of participants  *(b) Cohort study* - For matched studies, give matching criteria and number of exposed and unexposed  *Case-control study* - For matched studies, give matching criteria and the number of controls per case |  | RECORD 6.1: The methods of study population selection (such as codes or algorithms used to identify subjects) should be listed in detail. If this is not possible, an explanation should be provided.  RECORD 6.2: Any validation studies of the codes or algorithms used to select the population should be referenced. If validation was conducted for this study and not published elsewhere, detailed methods and results should be provided.  RECORD 6.3: If the study involved linkage of databases, consider use of a flow diagram or other graphical display to demonstrate the data linkage process, including the number of individuals with linked data at each stage. | Methods |
| Variables | 7 | Clearly define all outcomes, exposures, predictors, potential confounders, and effect modifiers. Give diagnostic criteria, if applicable. |  | RECORD 7.1: A complete list of codes and algorithms used to classify exposures, outcomes, confounders, and effect modifiers should be provided. If these cannot be reported, an explanation should be provided. | Methods |
| Data sources/ measurement | 8 | For each variable of interest, give sources of data and details of methods of assessment (measurement).  Describe comparability of assessment methods if there is more than one group |  |  | Supplement |
| Bias | 9 | Describe any efforts to address potential sources of bias |  |  | Methods |
| Study size | 10 | Explain how the study size was arrived at |  |  | Methods and Protocol |
| Quantitative variables | 11 | Explain how quantitative variables were handled in the analyses. If applicable, describe which groupings were chosen, and why |  |  | Supplement |
| Statistical methods | 12 | (a) Describe all statistical methods, including those used to control for confounding  (b) Describe any methods used to examine subgroups and interactions  (c) Explain how missing data were addressed  (d) *Cohort study* - If applicable, explain how loss to follow-up was addressed  *Case-control study* - If applicable, explain how matching of cases and controls was addressed  *Cross-sectional study* - If applicable, describe analytical methods taking account of sampling strategy  (e) Describe any sensitivity analyses |  |  | Methods |
| Data access and cleaning methods |  | .. |  | RECORD 12.1: Authors should describe the extent to which the investigators had access to the database population used to create the study population.  RECORD 12.2: Authors should provide information on the data cleaning methods used in the study. | Methods and Supplement |
| Linkage |  | .. |  | RECORD 12.3: State whether the study included person-level, institutional-level, or other data linkage across two or more databases. The methods of linkage and methods of linkage quality evaluation should be provided. | Methods |
| Results | | | | | |
| Participants | 13 | (a) Report the numbers of individuals at each stage of the study (*e.g.*, numbers potentially eligible, examined for eligibility, confirmed eligible, included in the study, completing follow-up, and analysed)  (b) Give reasons for non-participation at each stage.  (c) Consider use of a flow diagram |  | RECORD 13.1: Describe in detail the selection of the persons included in the study (*i.e.,* study population selection) including filtering based on data quality, data availability and linkage. The selection of included persons can be described in the text and/or by means of the study flow diagram. | Methods |
| Descriptive data | 14 | (a) Give characteristics of study participants (*e.g.*, demographic, clinical, social) and information on exposures and potential confounders  (b) Indicate the number of participants with missing data for each variable of interest  (c) *Cohort study* - summarise follow-up time (*e.g.*, average and total amount) |  |  | Results |
| Outcome data | 15 | *Cohort study* - Report numbers of outcome events or summary measures over time  *Case-control study* - Report numbers in each exposure category, or summary measures of exposure  *Cross-sectional study* - Report numbers of outcome events or summary measures |  |  | Results |
| Main results | 16 | (a) Give unadjusted estimates and, if applicable, confounder-adjusted estimates and their precision (e.g., 95% confidence interval). Make clear which confounders were adjusted for and why they were included  (b) Report category boundaries when continuous variables were categorized  (c) If relevant, consider translating estimates of relative risk into absolute risk for a meaningful time period |  |  | Results |
| Other analyses | 17 | Report other analyses done—e.g., analyses of subgroups and interactions, and sensitivity analyses |  |  | Supplement |
| Discussion | | | | | |
| Key results | 18 | Summarise key results with reference to study objectives |  |  | Discussion |
| Limitations | 19 | Discuss limitations of the study, taking into account sources of potential bias or imprecision. Discuss both direction and magnitude of any potential bias |  | RECORD 19.1: Discuss the implications of using data that were not created or collected to answer the specific research question(s). Include discussion of misclassification bias, unmeasured confounding, missing data, and changing eligibility over time, as they pertain to the study being reported. | Limitations |
| Interpretation | 20 | Give a cautious overall interpretation of results considering objectives, limitations, multiplicity of analyses, results from similar studies, and other relevant evidence |  |  | Conclusion |
| Generalisability | 21 | Discuss the generalisability (external validity) of the study results |  |  | Discussion |
| Other Information | | | | | |
| Funding | 22 | Give the source of funding and the role of the funders for the present study and, if applicable, for the original study on which the present article is based |  |  | Funding |
| Accessibility of protocol, raw data, and programming code |  | .. |  | RECORD 22.1: Authors should provide information on how to access any supplemental information such as the study protocol, raw data, or programming code. | Data Sharing note |

# Supplement 9: Primary, secondary and sensitivity analyses

Table S33 – Primary (P), secondary (S) and sensitivity (Sn) analyses detailing match ratio, matching algorithm and whether the block matching was undertaken for both sites or was site specific. All analyses were undertaken in Stata v18. ^15^

|  |  |  |  | Site | HR | 95% CI | Patients, n | | ED attendances, n | |
| --- | --- | --- | --- | --- | --- | --- | --- | --- | --- | --- |
|  |  |  |  |  |  |  | Control | Intervention | Control | Intervention |
| P | CEM | 1:M | PP | Both | 0.95 | 0.91 - 0.99 | 6,196 | 2,068 | 12,174 | 3,580 |
| S | CEM | 1:M | PP_r_ | Both | 0.99 | 0.94 - 1.04 | 5,142 | 1,226 | 9,699 | 2,096 |
| S | CEM | 1:M | ITT | Both | 0.97 | 0.94 - 0.99 | 8,926 | 5,963 | 17,919 | 10,304 |
| Sn | CEM | 1:M | PP | Cardiff | 0.93 | 0.89 - 0.97 | 5,787 | 1,783 | 11,749 | 3,226 |
| Sn | CEM | 1:M | PP_r_ | Cardiff | 0.96 | 0.91 - 1.01 | 4,547 | 972 | 9,170 | 1,789 |
| Sn | CEM | 1:M | ITT | Cardiff | 0.94 | 0.91 - 0.97 | 8,076 | 4,135 | 16,927 | 7,957 |
| Sn | CEM | 1:M | PP | Swansea | 1.18 | 1.05 - 1.33 | 1,472 | 282 | 1,506 | 344 |
| Sn | CEM | 1:M | PP_r_ | Swansea | 1.23 | 1.09 - 1.39 | 1,406 | 254 | 1,400 | 307 |
| Sn | CEM | 1:M | ITT | Swansea | 1.07 | 1.02 - 1.12 | 4,352 | 1,828 | 5,289 | 2,347 |
| Sn | PSM | 1:1 | PP | Both | 0.91 | 0.88 - 0.95 | 2,519 | 2,518 | 5,601 | 5,080 |
| Sn | PSM | 1:1 | PP_r_ | Both | 0.93 | 0.89 - 0.98 | 1,499 | 1,498 | 3,191 | 2,973 |
| Sn | PSM | 1:1 | ITT | Both | 0.99 | 0.97 - 1.02 | 6,687 | 6,683 | 13,555 | 13,404 |
| Sn | PSM | 1:1 | PP | Cardiff | 0.92 | 0.88 - 0.96 | 2,148 | 2,147 | 4,984 | 4,547 |
| Sn | PSM | 1:1 | PP_r_ | Cardiff | 0.93 | 0.88 - 0.98 | 1,171 | 1,170 | 2,647 | 2,489 |
| Sn | PSM | 1:1 | ITT | Cardiff | 0.97 | 0.94 - 0.99 | 4,777 | 4,773 | 10,895 | 10,448 |
| Sn | PSM | 1:1 | PP | Swansea | 1.10 | 0.97 - 1.25 | 367 | 367 | 457 | 526 |
| Sn | PSM | 1:1 | PP_r_ | Swansea | 1.20 | 1.05 - 1.37 | 327 | 327 | 385 | 472 |
| Sn | PSM | 1:1 | ITT | Swansea | 1.00 | 0.95 - 1.05 | 2,228 | 2,228 | 3,248 | 3,273 |
| Sn | CEM | 1:1 | PP | Both | 0.95 | 0.90 - 0.99 | 1,984 | 1,983 | 3,732 | 3,482 |
| Sn | CEM | 1:1 | PP_r_ | Both | 1.04 | 0.97 - 1.10 | 1,201 | 1,200 | 1,976 | 2,043 |
| Sn | CEM | 1:1 | ITT | Both | 0.94 | 0.91 - 0.97 | 5,117 | 5,115 | 9,737 | 9,056 |
| Sn | CEM | 1:1 | PP | Cardiff | 0.93 | 0.89 - 0.98 | 1,717 | 1,716 | 3,435 | 3,147 |
| Sn | CEM | 1:1 | PP_r_ | Cardiff | 0.98 | 0.92 - 1.04 | 953 | 952 | 1,828 | 1,770 |
| Sn | CEM | 1:1 | ITT | Cardiff | 0.94 | 0.91 - 0.97 | 3,817 | 3,815 | 7,951 | 7,380 |
| Sn | CEM | 1:1 | PP | Swansea | 1.20 | 1.03 - 1.40 | 280 | 280 | 292 | 340 |
| Sn | CEM | 1:1 | PP_r_ | Swansea | 1.18 | 1.00 - 1.39 | 253 | 253 | 260 | 306 |
| Sn | CEM | 1:1 | ITT | Swansea | 1.12 | 1.05 - 1.18 | 1,739 | 1,739 | 1,995 | 2,230 |
| Sn | PSM | 1:M | PP | Both | 0.97 | 0.94 - 1.01 | 13,668 | 2,537 | 32,087 | 5,109 |
| Sn | PSM | 1:M | PP_r_ | Both | 0.97 | 0.92 - 1.02 | 13,668 | 1,503 | 32,087 | 2,983 |
| Sn | PSM | 1:M | ITT | Both | 0.97 | 0.95 - 1.00 | 13,670 | 7,186 | 32,087 | 13,984 |
| Sn | PSM | 1:M | PP | Cardiff | 0.99 | 0.95 - 1.03 | 13,670 | 2,169 | 32,087 | 4,584 |
| Sn | PSM | 1:M | PP_r_ | Cardiff | 0.97 | 0.92 - 1.03 | 13,669 | 1,175 | 32,087 | 2,504 |
| Sn | PSM | 1:M | ITT | Cardiff | 0.96 | 0.94 - 0.99 | 13,670 | 4,958 | 32,087 | 10,711 |
| Sn | PSM | 1:M | PP | Swansea | 1.01 | 0.89 - 1.14 | 7,746 | 369 | 11,374 | 528 |
| Sn | PSM | 1:M | PP_r_ | Swansea | 1.05 | 0.93 - 1.20 | 6,826 | 327 | 9,259 | 472 |
| Sn | PSM | 1:M | ITT | Swansea | 1.04 | 0.99 - 1.10 | 7,752 | 2,228 | 11,383 | 3,273 |

Results for the primary analysis CEM PP 1:M the hazard rate is broadly consistent with 1:1 and PSM for both sites, except for PSM 1:M. The latter null result is likely attributable to the PSM algorithm introducing residual imbalance. ^8^ Restricting to patients with a minimum 12-month follow-up duration further replicates these results (Table S34).

##### Table S34 – Primary (P) and secondary (S) analyses restricting the sample to patients with a minimum 12-month follow-up, detailing match ratio, matching algorithm and whether the block matching was undertaken for both sites or was site specific. All analyses were undertaken in Stata v18.

|  |  |  |  | Site | HR | 95% CI | Patients, n | | ED attendances, n | |
| --- | --- | --- | --- | --- | --- | --- | --- | --- | --- | --- |
|  |  |  |  |  |  |  | Control | Intervention | Control | Intervention |
| P | CEM | 1:M | PP | Both | 0.94 | 0.90 to 0.98 | 5,197 | 1,710 | 11,628 | 3,350 |
| S | CEM | 1:M | PP_r_ | Both | 0.97 | 0.92 to 1.02 | 4,259 | 988 | 9,181 | 1,914 |
| S | CEM | 1:M | ITT | Both | 0.96 | 0.93 to 0.98 | 7,417 | 4,814 | 17,007 | 9,510 |

##### Table S35 – Descriptive statistics for the treatment group, by their extent of engagement in the intervention.

|  | Not Engaged | Engaged |
| --- | --- | --- |
| N | 3,895 | 2,068 |
| Gender |  |  |
| Female | 1337 (34.33%) | 545 (26.35%) |
| Male | 2558 (65.67%) | 1,523 (73.65%) |
| Ethnic Group |  |  |
| White | 3186 (81.8%) | 1,562 (75.53%) |
| Mixed or Multiple | 46 (1.18%) | 35 (1.69%) |
| Asian | 130 (3.34%) | 106 (5.13%) |
| Black | 28 (0.72%) | 17 (0.82%) |
| Other ethnic group | 479 (12.3%) | 335 (16.2%) |
| Missing | 26 (0.67%) | 13 (0.63%) |
| Age Group |  |  |
| Age 11 to 17 Years | 486 (12.48%) | 651 (31.48%) |
| Age 18 to 30 Years | 1590 (40.82%) | 695 (33.61%) |
| Age 31 to 50 Years | 1438 (36.92%) | 550 (26.6%) |
| Age 51+ Years | 381 (9.78%) | 172 (8.32%) |
| Admitted into Hospital |  |  |
| No | 3823 (98.15%) | 2,030 (98.16%) |
| Admitted | 72 (1.85%) | 38 (1.84%) |
| Residence |  |  |
| Rural | 262 (6.73%) | 107 (5.17%) |
| Urban | 3633 (93.27%) | 1,961 (94.83%) |
| Quintile of Deprivation |  |  |
| Most Deprived | 1688 (43.34%) | 963 (46.57%) |
| 2 | 779 (20.00%) | 367 (17.75%) |
| 3 | 551 (14.15%) | 274 (13.25%) |
| 4 | 372 (9.55%) | 215 (10.4%) |
| Least Deprived | 505 (12.97%) | 249 (12.04%) |
| Previous Month ED Attendance |  |  |
| None | 3764 (96.64%) | 1,958 (94.68%) |
| One or More | 131 (3.36%) | 110 (5.32%) |

# Supplement 10: Interactions

Table S36 – Treatment covariate interactions

|  |  | HR (95% CI) |
| --- | --- | --- |
| Allocation | |  |
|  | Control | Reference |
|  | Intervention | 0.58 (0.46-0.73) |
| Gender | |  |
|  | Female | Reference |
|  | Male | 0.73 (0.70-0.76) |
| Age | |  |
|  | Age 11 to 17 Years | Reference |
|  | Age 18 to 30 Years | 1.00 (0.96-1.05) |
|  | Age 31 to 50 Years | 1.09 (1.04-1.15) |
|  | Age 50+ Years | 0.81 (0.75-0.88) |
| Ethnicity | |  |
|  | White: English, Welsh, Scottish, Northern Irish or British | Reference |
|  | Mixed or Multiple | 1.08 (0.94-1.24) |
|  | Asian: Asian British or Asian Welsh, Bangladeshi | 0.67 (0.56-0.80) |
|  | Black: Black British, Black Welsh, Caribbean or African, African | 0.86 (0.68-1.08) |
|  | Other | 0.78 (0.71-0.85) |
|  | Missing | 2.34 (1.92-2.85) |
| Discharge | |  |
|  | Home | Reference |
|  | Admitted | 0.74 (0.64-0.85) |
| Residence | |  |
|  | Rural | Reference |
|  | Urban | 1.00 (0.90-1.10) |
| Deprivation | |  |
|  | Least Deprived Quintile | Reference |
|  | 2nd Quintile | 0.91 (0.87-0.96) |
|  | 3rd Quintile | 0.75 (0.71-0.80) |
|  | 4th Quintile | 0.73 (0.68-0.78) |
|  | Most Deprived Quintile | 0.80 (0.75-0.85) |
| Previous Month Attendance | |  |
|  | No ED Attendance | Reference |
|  | One or more ED attendances | 2.49 (2.36-2.63) |
| Characteristic - Treatment Interactions | | |
| Gender x Allocation | |  |
|  | Male | 1.18 (1.09-1.29) |
| Age Group x Allocation | |  |
|  | Age 18 to 30 Years | 0.96 (0.87-1.06) |
|  | Age 31 to 50 Years | 1.35 (1.22-1.48) |
|  | Age 50+ Years | 1.27 (1.09-1.50) |
| Ethnicity x Allocation | |  |
|  | Mixed or Multiple | 0.80 (0.60-1.08) |
|  | Asian | 1.04 (0.81-1.33) |
|  | Black | 0.96 (0.59-1.56) |
|  | Other ethnic group | 1.01 (0.89-1.15) |
|  | Missing | 0.31 (0.16-0.59) |
| Discharge x Allocation | |  |
|  | Admitted | 1.20 (0.91-1.58) |
| Residence x Allocation | |  |
|  | Urban | 1.38 (1.11-1.71) |
| Deprivation x Allocation | |  |
|  | 2nd Quintile | 1.08 (0.98-1.19) |
|  | 3rd Quintile | 1.15 (1.01-1.31) |
|  | 4th Quintile | 1.27 (1.11-1.46) |
|  | Most Deprived Quintile | 0.98 (0.86-1.12) |
| Previous Month Attendance x Allocation | |  |
|  | One or more ED attendances | 0.75 (0.67-0.84) |

For the above interactions, the HR on, for example, male (HR = 1.18, 95% CI 1.09 to 1.29) should be interpreted as the difference in HRs between males and females, holding females at HR = 1 (Figure S1). Therefore, the Gender x Allocation suggests women, compared to men, are more likely to benefit from the intervention. The Age Group x Allocation suggests that patients aged 11 to 30 years of age are relatively more likely to benefit from the intervention. Patients not admitted into hospital at discharge were more likely to benefit compared to those who were admitted into hospital. Those living in a more rural location were more likely to benefit compared to those in an urban location. Those living in the less deprived neighbourhoods were more likely to benefit from the intervention, accepting the most deprived quintile which is for the null and likely attributable to smaller numbers in this group. Notably, those with one or more ED attendances in the previous month were more likely to benefit, compared to those without a recent attendance, suggesting that the intervention might benefit more frequent ED attenders. Stata v18 lincom was used to estimate the HR for each of age, gender and previous month attendance (Table S36).


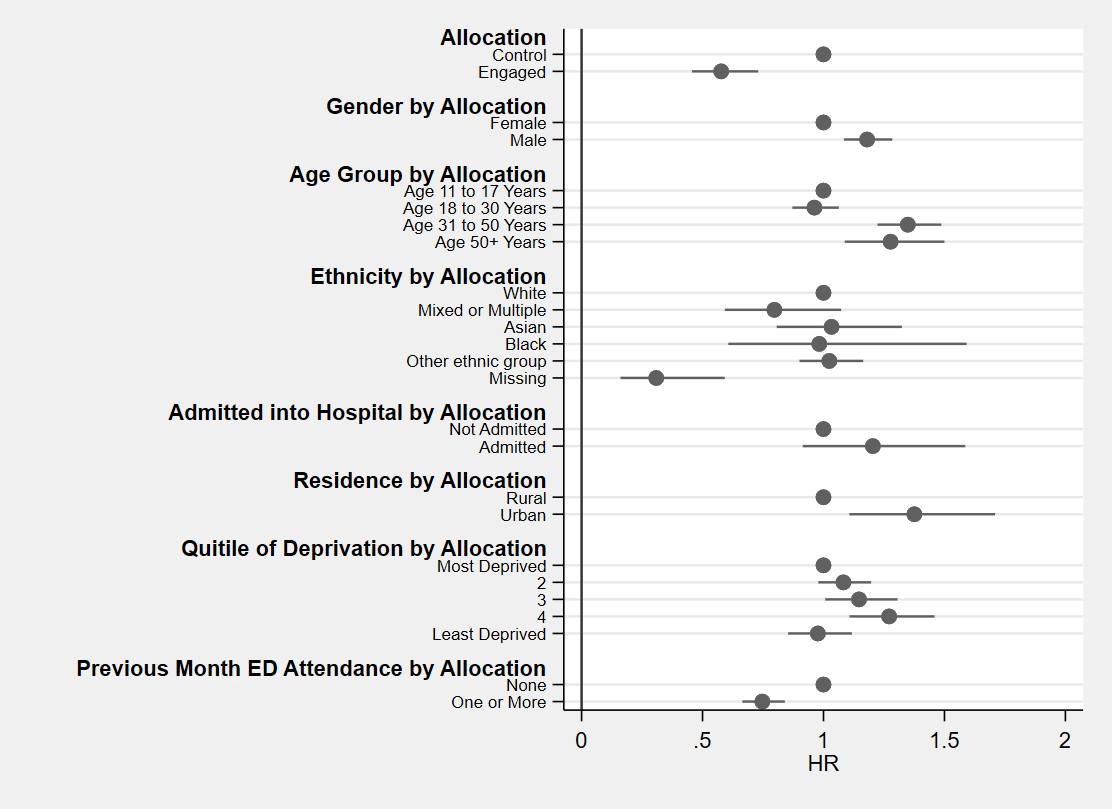


Figure S1 – Graphical representation of interactions

Table S37 – Estimated effects of the intervention on hazard ratios for age, gender and previous month ED attendance

|  |  | HR | 95% CI |
| --- | --- | --- | --- |
| Gender |  |  |  |
|  | Female | 0.86 | 0.80 to 0.92 |
|  | Male | 1.04 | 0.99 to 1.08 |
| Age (years) |  |  |  |
|  | Age 11-17 | 0.88 | 0.82 to 0.92 |
|  | Age 18-30 | 0.86 | 0.80 to 0.92 |
|  | Age 31-50 | 1.19 | 1.12 to 1.27 |
|  | Age 50+ | 1.17 | 1.07 to 1.35 |
| Previous Month Attendance |  |  |  |
|  | None | 1.02 | 0.98 to 1.06 |
|  | One or more | 0.77 | 0.69 to 0.86 |

# References

1. Hoffmann TC, Glasziou PP, Boutron I, et al. Better reporting of interventions: template for intervention description and replication (TIDieR) checklist and guide. *Bmj* 2014;348

2. Newbury A. A service evaluation of the delivery and implementation of a hospital-based Violence Prevention Team within the University Hospital of Wales Cardiff, UK: Public Health Wales; 2022 [Available from: <https://www.violencepreventionwales.co.uk/cms-assets/research/A-Service-Evaluation-of-the-Delivery-and-Implementation-of-a-Hospital-Based-Violence-Prevention-Team-within-the-University-Ho.pdf> accessed September 2025.

3. Ford DV, Jones KH, Verplancke J-P, et al. The SAIL Databank: building a national architecture for e-health research and evaluation. *BMC Health Services Research* 2009;9(1):1–12.

4. Lyons RA, Jones KH, John G, et al. The SAIL databank. *BMC Medical Informatics and Decision Making* 2009;9(1):1–8.

5. Jones KH, Ford DV, Jones C, et al. A case study of the Secure Anonymous Information Linkage (SAIL) Gateway: a privacy-protecting remote access system for health-related research and evaluation. *Journal of biomedical informatics* 2014;50:196–204.

6. King G, Nielsen R. Why propensity scores should not be used for matching. *Political analysis* 2019;27(4):435–54.

7. King G, Nielsen R, Coberley C, et al. Comparative effectiveness of matching methods for causal inference. *Unpublished manuscript, Institute for Quantitative Social Science, Harvard University, Cambridge, MA* 2011

8. Imbens GW. Matching methods in practice: Three examples. *Journal of Human Resources* 2015;50(2):373–419.

9. Iacus SM, King G, Porro G. Causal inference without balance checking: Coarsened exact matching. *Political analysis* 2012;20(1):1–24.

10. Sturges HA. The choice of a class interval. *Journal of the american statistical association* 1926;21(153):65–66.

11. Guo Z, Gill TM, Allore HG. Modeling repeated time-to-event health conditions with discontinuous risk intervals. *Methods of information in medicine* 2008;47(02):107–16.

12. Rücker G, Messerer D. Remission duration: an example of interval‐censored observations. *Statistics in Medicine* 1988;7(11):1139–45.

13. Staniszewska S, Brett J, Simera I, et al. GRIPP2 reporting checklists: tools to improve reporting of patient and public involvement in research. *bmj* 2017;358

14. Benchimol EI, Smeeth L, Guttmann A, et al. The REporting of studies Conducted using Observational Routinely-collected health Data (RECORD) statement. *PLoS medicine* 2015;12(10):e1001885.

15. Stata Statistical Software: Release 18 [program]. College Station, TX: StataCorp LLC, 2024.
