# Supplementary material for: A quasi-experimental effectiveness evaluation of a nurse-led Hospital Violence Intervention Programme in the United Kingdom using linked routine health and administrative data
Source: eClinicalMedicine. 2026 Mar 26;94:103848. doi: 10.1016/j.eclinm.2026.103848 (PMC13054530; doi:10.1016/j.eclinm.2026.103848)
Supplement: EDVIPE_protocol [file mmc2.pdf]

Protocol

Effectiveness and Cost-Effectiveness of Emergency Department–Based Violence Intervention Programs in the United Kingdom: Protocol for a Quasi-Experimental Study

Simon Moore[1,2], PhD; Sinead Brophy[3], PhD; Amrita Bandyopadhyay[3], PhD; Annemarie Newbury[1], MSc; Megan Hamilton[1], MSc; Adele Battaglia[1], MA Ed; Trudy Lowe[2], MSc; David O'Reilly[4], MD; David Rawlinson[5], MRes; Lara Snowdon[6], MSc; Jonathan Shepherd[1,2], DDSc; Vaseekaran Sivarajasingam[1], PhD; Alan Watkins[7], PhD; Simon Walker[8], MSc; Shainur Premji[8], PhD; Sophie Borgia[1], MSc; Henry Yeomans[1], MSc

[1]Violence Research Group, School of Dentistry, Cardiff University, Cardiff, United Kingdom
[2]Security, Crime and Intelligence Innovation Institute, Cardiff University, Cardiff, Wales, United Kingdom
[3]Health Data Science, Swansea University, Swansea, United Kingdom
[4]Cardiff Liver Unit, School of Medicine, University Hospital of Wales, Cardiff, United Kingdom
[5]Emergency Medical Retrieval & Transfer Service, Wales Air Ambulance, Tŷ Elusen, Ffordd Angel, Llanelli Gate, Dafen, United Kingdom
[6]Public Health Wales, Cardiff, United Kingdom
[7]Medical School, Swansea University, Swansea, United Kingdom
[8]Centre for Health Economics, University of York, York, United Kingdom

Corresponding Author:
Simon Moore, PhD
Violence Research Group
School of Dentistry
Cardiff University
Heath Park
Cardiff, CF14 4XY
United Kingdom
Phone: 1 44 2920874000
Email: mooresc2@cardiff.ac.uk

Abstract

Background: Hospital-Based Violence Intervention Programs (HVIPs), based in Emergency Departments (EDs), have been proposed as a public health response to violence. These programs address the underlying reasons why patients are exposed to violence. In addressing any underlying modifiable risks and vulnerabilities HVIPs can reduce patients' exposure to violence and therefore subsequent unplanned attendance into ED.

Objective: The objectives of this study are to (1) assess whether patient involvement with a HVIP reduces the likelihood of unscheduled ED reattendance, (2) determine whether the presence of the HVIP improves ascertainment of violence in ED attendances, and (3) derive the costs of the HVIP and compare those to the benefits of the intervention and understand whether the HVIP represents value for money from a health service perspective. If an effect is observed, then models will estimate the health impacts, costs and potential savings over a longer time (eg, 10 years) period and for a national roll-out.

Methods: ED patients are eligible for inclusion in the evaluation if they are normally resident in Wales, United Kingdom, aged 11 years and older. A controlled longitudinal natural experiment will be undertaken. The primary outcome is derived from the Emergency Department Dataset, routinely collected for all EDs in Wales, and is subsequent unplanned ED attendance. Case patients will be matched to control patients attending EDs without an HVIP. Analysis will derive the hazard rate for subsequent unplanned ED attendances using recurrent event analysis. The total monthly count of patients identified as attending because of violence in intervention EDs will be compared to the total count of Welsh control EDs in an interrupted time-series analysis to determine whether HVIPS increase violence ascertainment. To determine whether referral, versus no referral, to the HVIP represents value for money, we will undertake a cost-effectiveness analysis from the perspective of the National Health Service. The approval to access and analyze data housed in the Secure Anonymized Information Linkage (SAIL) databank, an ISO (International Organization for Standardization) 27001 certified and UK Statistics Authority accredited secure data environment,

XSL•FO
RenderX

was granted by the SAIL independent Information Governance Review Panel (Ref: 1421). Findings will be presented at local, national, and international conferences and disseminated by peer-reviewed publication.

Results: Design inputs arising from public patient involvement and engagement (PPIE) are reported. As a protocol, no further results are available.

Conclusions: Novel methods are developed to provide the first robust evaluation of Emergency Department Violence Intervention Programs (EDVIPs).

Trial Registration: ISRCTN Registry ISRCTN68945844; https://www.isrctn.com/ISRCTN68945844?q=68945844&filters=&sort=&offset=1&totalResults=1&page=1&pageSize=10

International Registered Report Identifier (IRRID): PRR1-10.2196/86247

(JMIR Res Protoc 2026;15:e86247) doi: 10.2196/86247

KEYWORDS

emergency department; assault; violence; injury; prevention; treatment

Introduction

Background

Those who experience serious injury due to violence are likely to attend an Emergency Department (ED). EDs are therefore ideal locations for Hospital-Based Violence Intervention Programs (HVIPs) [1-3]. HVIPs have recently emerged as a public health response to violent victimization [3,4], but despite interest in HVIPs, there has been no rigorous evaluation of this public health approach to violence in the United Kingdom. Moreover, little is known about the effectiveness of patient discharge planning and referral from ED into organizations able to support children involved in violence [5] and patients exposed to domestic violence [6,7], and there is a paucity of studies considering referrals for young men involved in violence, the most dominant population in respect of Assault-Related Attendance (ARA) [8]. There are even fewer studies of the effective support available to victims of sexual violence attending ED [9]. Despite uncertainty over effectiveness in a UK context, HVIPs have begun to be implemented. For example, the Scottish Violence Reduction Unit (VRU) has placed navigators in EDs, typically youth workers who connect with patients aged 25 years and younger [10].

EDs work with patients attending for many reasons, including those presenting with violence-related injuries. EDs also engage in broader violence reduction initiatives, clinical staff typically receive training in adult and pediatric safeguarding, and many EDs have provisions to identify and refer children and victims of domestic and sexual violence. However, methods of ascertainment and referral vary considerably, and formal relationships with the police and other partners can often lack continuity, with multiagency approaches not capturing the entirety of patients' journeys. A public health approach involving the ED would be beneficial in improving overall population health outcomes. It is particularly timely; in 2019 there were 175,764 ARAs at EDs in England and Wales [11] and, despite the pandemic, 119,111 ARAs in 2020 [12]. Knife crime, and therefore serious trauma, has risen by 71% from 2014 to 2018 [13]. Up to 75% of ARAs are unknown to the police [14-16]; therefore, EDs hold exclusive data on assault characteristics, patient vulnerabilities, and modifiable risks and are therefore well situated to play a significant role in the identification of violence, to investigate the circumstances of violence, and to challenge any underlying vulnerabilities or modifiable risks exposing patients to violence, whether that is through direct support, referral, or discharge planning.

The need for HVIPs is aligned to broader UK Government initiatives, which aim to promote a whole system multiagency (WSMA) [17] approach to violence. The 1998 Crime and Disorder Act requires the police, local government, and the National Health Service (NHS) to collaborate on joint crime reduction strategies, and this includes data sharing to inform targeted responses. Violence reduction is further prioritized by the UK Government in its serious violence strategy [18], and the UK Government has allocated funds for the formation of VRUs in England and a violence prevention unit (VPU) in Wales, across 18 Police and Crime Commissioner jurisdictions, with the explicit purpose of promoting the WSMA approach [19]. These initiatives are further aligned with a move toward active population health management, digitally enabled whole-person care, and evidence-based treatment pathways outlined in the NHS future plan [20]. Integrated care systems in NHS England will be expected to specify violence prevention and reduction standards, which are incorporated into the 2021/22 NHS Standard Contract, and there are expectations that hubs will form Violence Prevention Teams (VPTs) similar to the police VRUs and VPU. Furthermore, a public sector duty on partnerships encouraging the prioritization of reducing serious violence has received royal assent as a part of the police, crime, sentencing, and courts bill. This legislation includes a serious violence duty, placing a statutory obligation on organizations to collaborate, communicate, and act.

The overarching aim of the work proposed here is a robust effectiveness and cost-effectiveness evaluation of ED-based VPTs. VPTs represent a formal collaboration between police and health care and embody the WSMA approach. To our knowledge, this is the first formal evaluation of a nurse-led, ED-based HVIP in the United Kingdom and will address significant gaps in current understanding of their effectiveness and thereby facilitate future aspirations for evidence-based referral pathways and discharge planning [20,21]. VPTs main function is to identify and support patients attending ED with assault-related injury. To facilitate this, they engage in broader pedagogical roles, increasing awareness of these patients' needs,

XSL•FO
RenderX

modifiable risks, and opportunities to identify and refer across the ED clinical environment.

Theoretical Framework

The theoretical motivation for a WSMA approach to HVIPs is that there are many modifiable risks and vulnerabilities that, in combination, determine an individual's exposure to violence and subsequently an ARA in an ED. Epidemiologically, these can be usefully described by shared circumstances that in turn signpost opportunities to modify risk or support patients' vulnerability, but responsibility can fall across organizations, including local government, health care, and criminal justice. Risks include the consumption of alcohol and other psychoactive substances [22-25]; criminal and/or sexual exploitation and homelessness [3,26]. Violence tends to be more prevalent in younger, socially disadvantaged groups [27-33], with male, socioeconomically deprived individuals being more likely to endure violence and experience assault-related injury. These characteristics further extend to personality features [34], including mental health status and learning disability, and neurodevelopmental disorders [35]. This complex interplay of factors that promote exposure to violence, and hence lead to an ARA, highlight the need for a WSMA approach. For example, an environment might become synonymous with violence through a process of homophile [36], whereby individuals with shared pursuits who are at risk of violence gather, for example, street drinkers and late-night drinking environments. Mitigation might include challenging reasons for frequenting such an environment, including alcohol and substance misuse counseling. Some environments might involve those who use violence to advance their interests, such as acquisitive crime, sexual assault, or sexual exploitation, in which case criminal justice or safeguarding processes to deter violence might be involved, along with support to victims. Chaotic or otherwise disadvantaged households in which domestic violence or harm to children arises might best be approached from a multiagency process such as the Multi-Agency Risk Assessment Committee (MARAC) and formal investigation (Section 47, Children Act 1989). EDs are primary agencies receiving those who have sustained a serious injury, including those who are motivated to bypass other agencies or whose assailant is motivated to ensure their victim avoids scrutiny.

Treatment for an ARA in ED aims to address symptoms (eg, injury) that may not necessarily characterize the underlying reasons for violence (eg, alcohol dependency), and staff do not always have the resources available to address such modifiable risks and vulnerabilities. However, without addressing them, the risk of repeat unscheduled ED attendance remains, including violence recidivism. For these reasons, services like VPTs that work within a WSMA approach to better understand reasons for ARA are required. Moreover, for those who are most vulnerable, ED may be the only realistic opportunity for patients to enter a system of care. As such, an ARA is often a sentinel event.

Aims and Objectives

The overarching aim of the Emergency Department Violence Intervention Program Evaluation (EDVIPE) is to determine whether VPTs are effective and cost-effective from the perspective of the NHS.

- Objective 1: to assess whether patient involvement with a VPT reduces the likelihood of unscheduled ED reattendance. We consider case and control patients' ED unscheduled reattendance for a minimum of 12 months following the initial ARA.
- Objective 2: to determine whether the presence of the VPT improves ascertainment of ARAs in ED attendances. We will consider the change in identified ED ARAs across intervention implementation in case and control EDs in Wales.
- Objective 3: to derive the costs of the VPT and compare those to the benefits of the intervention and understand whether the VPT represents value for money from an NHS perspective. If an effect is observed, then models will estimate the health impacts, costs, and potential savings over a longer time period (eg, 10 years) and for a national rollout.

Methods

Intervention

A process and implementation evaluation that describes both the planned and implemented VPT intervention is available elsewhere [37] and is further described in a Template for Intervention Description and Replication (TIDieR; Multimedia Appendix 1 [38]).

Intervention as Hypothesized

VPTs, which emerged from the VPU violence prevention strategy, were funded by the UK Home Office and Youth Endowment Fund (YEF), with the funding administered by the VPU and the Office of the South Wales Police and Crime Commissioner (Multimedia Appendices 2 and 3). Other HVIPs in the United Kingdom are volunteer-based, whereas the VPTs are nurse-led. The original implementation for VPTs was focused on identifying and supporting ED patients aged 11-25 years and on formalizing the identification of modifiable risks and vulnerabilities, supporting and advising patients, and to signposting to other services as appropriate. The VPTs also aimed to raise awareness of the service across ED clinical teams, with the aim of it becoming embedded within usual practice, and to train and upskill the clinical team to enable ascertainment and referral.

Intervention as Implemented

Since November 2019, a collaborative VPT between the police and NHS has been operational in a South Wales Type I (consultant-led with resus) ED in Cardiff (the capital and largest city in Wales), and a second VPT began in an adjacent South Wales Type I ED in Swansea (the second-largest city in Wales) in January 2022. The VPTs initially sought to identify patients attending the ED due to violence. This remit was broadened, with VPTs subsequently receiving referrals from across the hospital and other community health care teams (eg, Minor Injury Units [MIUs] and General Practitioners).

XSL•FO
RenderX

The VPTs work with patients to gain an understanding of any circumstances contributing to their exposure to violence. They refer patients into care pathways (primary, secondary, and tertiary care, or third-sector organizations) to address any vulnerabilities or modifiable risks and can work alongside the third sector (nonprofit and charitable enterprise) to provide continual case management. The VPTs also train other staff within the hospital to improve the identification of violence-related injury, to support clinical staff interactions with patients, and to maintain safeguarding procedures. In addition, the EDs at Cardiff and Swansea take part in information sharing to tackle violence, in which anonymous data and intelligence regarding violent incidents are shared with community safety partnerships. These anonymized data enable partner resources to be best used for violence prevention, part of the Cardiff Model for violence prevention [39]. Following implementation, both VPTs expanded the age range of patients to encompass all age groups.

Usual Care

Under usual practice, clinical ED staff are obliged to undertake safeguarding activities and provide for those attending due to violence. However, provision varies across EDs. Under usual practice, when people attend ED with an injury suspected to be caused by violence, their injuries are treated, and the patient is encouraged to contact the police or have the ED contact the police on their behalf. In cases of serious injury involving weapon use, the ED is obliged to contact the police irrespective of patient consent. In terms of general safeguarding, all patient-facing clinical staff are expected to have up-to-date safeguarding training and thus to carry out safeguarding tasks. Patients who are experiencing domestic violence can be referred to an Independent Domestic Violence Advocate (IDVA). Patients attending due to sexual assault can be referred to an Independent Sexual Violence Advocate (ISVA). For the 10 control EDs in Wales, 2 EDs have an IDVA, and 2 others have access to an IDVA not based in their ED. Furthermore, ED staff can also refer patients in a MARAC, typically in cases where the criteria are not met for formal safeguarding but the clinician suspects that something is not right. To facilitate, multiagency referral forms are filled out for children who require safeguarding, and Vulnerable Adult VA1 Forms (to support the referral of vulnerable adults) are completed for vulnerable adults who require safeguarding.

There are IDVAs based in the intervention EDs in Cardiff and Swansea. Only Cardiff has an ISVA. Broadly, usual practice focuses on children and victims of domestic violence. The patients eligible for VPT support are therefore those who are not eligible for support from the IDVA or ISVA and are typically older than 10 years. Apart from the IDVAs in control EDs, none have additional resources specifically dedicated to the role of supporting patients attending due to violence, relying mainly on existing clinical staff to support safeguarding within their departments. This may involve naming an existing member of staff as a safeguarding ambassador or having a nurse act as safeguarding lead for the department. Across Welsh EDs, some control EDs have provisions for victims of domestic violence that includes cards with the “Live Fear Free” helpline that they can give to patients experiencing domestic violence and who do not meet the criteria for a MARAC referral. Similarly, none of the control EDs have processes in place to support patients’ referral to outside agencies. If patients disclose that they are struggling with issues, or if staff suspect patients are experiencing an issue, then referrals will be made by clinical staff. However, this is not the same as VPT members working with patients to identify modifiable risks and vulnerabilities that contribute to their experience of violence. The VPTs in Cardiff and Swansea are therefore unique.

Design and Conceptual Framework

A controlled longitudinal whole population (Wales, United Kingdom) natural experiment. The intervention in Cardiff began in November 2019, and in January 2022 in Swansea. Due to earlier changes in Emergency Department Data Set (EDDS) coding, these data are consistent and available from January 2012. Intervention data collection therefore begins in November 2019 and ends in August 2023, allowing a 12-month follow-up of patients until August 2024.

Objective 1: Effectiveness

We hypothesize that engagement with the VPT will help patients overcome modifiable risks and receive support for vulnerabilities, and that therefore the intervention will reduce the recurrence of unscheduled ED attendance.

Other than those who are most seriously injured, patients will register at ED reception and be triaged, at which point the most appropriate pathway through ED will be determined. At reception patients will be asked about the reason for their attendance, including whether it was due to an assault, data that becomes a part of the Patient Management System (PMS) [39]. Patients may not disclose that the reason for their attendance was assault-related. They might be reluctant, the perpetrator may have accompanied them, or they may wish to avoid scrutiny. One function of the VPTs is to work across clinical teams to improve ascertainment of ARAs. The result is that patients can be stratified according to the extent that they engage with the intervention.

1. Patients identified in the ED PMS data as having attended due to an assault, but with no further contact with the VPT.
2. Patients identified in the ED PMS or VPT data as having attended due to an assault but did not further engage with the VPT.
3. Patients identified in the ED PMS and VPT data and who engaged with the VPT.

The primary analysis concerns group 3. The reasons for patients not engaging are potentially related to underlying characteristics and in secondary analyses we will explore this. However, it is reasonable to assume that the 3 groups represent varying levels of intervention dose, and therefore secondary analyses and analyses using groups 1-3 will be informative.

Objective 2: Ascertainment

Our second hypothesis is that intervention implementation improves ARA ascertainment.

Patients attending ED can do so repeatedly within periods of time. This frequency is likely associated with the modifiable

XSL•FO
RenderX

risks associated with ARA and is of interest here. For Objective 2 it is therefore appropriate to determine the proportion of attendances identified as violence related. This generates time-series data. The outcome of interest is therefore the count of ARAs across all EDs. ARA attendance is defined as ARA in the ED PMS or, in the case of intervention sites, in the ED, PMS, or VPT data. Codes indicating the Provider Site (the ED in its hospital) are available in the EDDS, and this allows comparison between intervention EDs and control EDs. While intervention EDs have been in continual service since before the time-series start dates, this is not so for all Type I EDs in Wales; there have been several changes, with some EDs closing and others opening or being modified to receive additional patients. This, coupled with the intervention sites located at two of the largest hospitals in Wales, reduces the scope for selecting matching control sites [40], leading to potentially important baseline differences in the interrupted time-series data [41]. The counterfactual will therefore be ARAs across all control Type I EDs.

Objective 3: Cost-Effectiveness

We aim to determine whether the VPT represents value for money. The primary outcome will be quality-adjusted life years, which will be estimated based on effectiveness estimates comparing ED attendance, reattendance, and any injuries received for those engaging in the VPT service relative to those who do not. Costs will be captured from an NHS perspective, reflecting the cost of the intervention but also other costs to the NHS due to referral. A secondary cost-effectiveness analysis will undertake a societal perspective, which will explore additional costs across social care, the police, and the third sector.

Secondary Analyses

We aim to co-produce study protocols with collaborators and Patient and Public Involvement and Engagement (PPIE) groups to provide opportunities for them to shape secondary and additional epidemiological analyses. This facilitates opportunities to realize and contribute to what is a rapidly changing policy area. One example is our inclusion of school attainment, exclusions, and attendance in analyses, which have been highlighted in these early discussions. VPUs and VRUs have made little headway working with the education system to challenge the causes of violence, and this has been identified as a priority [42]. Furthermore, additional exploration is planned to characterize those excluded from the intervention but who have available Anonymous Linkage Fields (ALFs).

Population and Data

This is a whole-population evaluation, including all residents of Wales, United Kingdom. Data are housed in the Secure Anonymized Information Linkage (SAIL) databank [43].

Data

Violence Prevention Team Data (Cardiff and Swansea)

Intervention sites record patient details (name, date of birth, and gender), NHS number, extent of engagement with the VPT, and whether any subsequent referral was made.

Administrative Data

Several administrative datasets are available to characterize patients and summarized in Textbox 1.

Table 1 summarizes key patient socioeconomic and demographic characteristics required to enable secondary analyses in relevant subgroups.

Textbox 1. Emergency Department Violence Intervention Program Evaluation (EDVIPE) administrate datasets.

The following routinely collected administrative and population datasets will be linked and analyzed to support the evaluation of the Emergency Department Violence Intervention Program Evaluation (EDVIPE).

Annual District Death Extract (ADDE):

Provides week of birth and date of death, used to describe left- and right-side censoring of study participants.

Outpatient Referral Dataset (OPRD):

Includes data on outpatient referrals from primary care, helping to characterize referral pathways to secondary care. This dataset captures clinical referrals from General Practitioners, general and community dental practitioners, Accident and Emergency Departments, walk-in services, and consultant-to-consultant referrals.

Patient Episode Data for Wales (PEDW):

Covers individuals domiciled in Wales and treated in Welsh and English National Health Service (NHS) Trusts. Includes inpatient and day-case activity data, with spells and episode-level information on hospital admissions, enabling tracking of service use related to an ARA.

Welsh Demographic Service Dataset (WDSD):

Includes all individuals registered with a Welsh General Practitioner and, through anonymization, enables identification of household groups.

Welsh Index of Multiple Deprivation (WIMD):

The Welsh Government's official measure of relative deprivation for small areas in Wales, based on 8 domains, including income, employment, health, and access to services. WIMD is typically grouped into quintiles and is included in several NHS datasets.

2011 and 2021 UK Census:

National censuses are conducted every 10 years that include key demographic information. Due to patient entry into and exit from the EDVIPE, some individuals may be missing from the 2011 Census (eg, born after 2011) or the 2021 Census (eg, died before 2021); therefore, data from both censuses are required.

XSL•FO
RenderX

Table 1. Emergency Department Violence Intervention Program Evaluation (EDVIPE) patient characteristics.

Characteristic	Source
Age (from Week of Birth [WoB])	Welsh Demographic Service (WDS)
Sex	WDS, 2011 and 2021 Census
Ethnicity	2011 and 2021 Census
Asian (Bangladeshi, Chinese, Indian, Pakistani, Other Asian)	
Black (Caribbean, African, Other Black)	
Mixed (White and Asian, White and Black African, White and Black Caribbean, Other Mixed, or Multiple ethnic groups)	
White (English, Welsh, Scottish, Northern Irish, British, Irish, Gypsy or Irish Traveller, Roma, or Other White)	
Other (Arab, Any other ethnic group)	
Quintile of residential deprivation	Welsh Index of Multiple Deprivation (WIMD)
Urban or rural residential classification	2011 and 2021 Census

Secondary Outcomes

To characterize the WSMA involvement of patients involved in the intervention, broader pathways will be explored across several related datasets, summarized in Textbox 2.

Textbox 2. Emergency Department Violence Intervention Program Evaluation (EDVIPE) secondary outcomes.

Children and Family Court Advisory and Support Service (CAFCASS) Wales family justice dataset includes information for residents of England and Wales on cases of divorce, private law, Family Law Act cases, public law, adoption, and other family law applications. It also includes information on marriage and divorce characteristics, the number of children involved in cases, and types of hearings.

Ministry of Justice (MoJ): Data First includes linked administrative datasets covering:

- Magistrates’ court defendant data
- Crown Court defendant data
- Criminal courts and prisons data
- Prisoner custodial journey data
- Family Court data

National Pupil Database (NPD) contains 4 broad categories of data: demographics, educational attainment, absence and exclusion, and information on children in need and looked-after children.

Police Data (Police Crime Dataset): Pending applications for police crime data from all 4 Welsh police forces are progressing and will be explored. Data sharing agreements are currently being developed to bring all-Wales police crime data into Secure Anonymized Information Linkage.

Substance Misuse Dataset (SMSD), also known as the Welsh National Database for Substance Misuse (WNDSM), includes data on individuals in Wales presenting for substance misuse treatment, including assessments, referrals, and treatment history.

Welsh Longitudinal General Practitioner Dataset (WLGPD) contains clinical information from general practices in Wales, including diagnoses and referrals to secondary and tertiary care.

Cohort Inclusion and Exclusion Criteria

All residents of Wales who are 11 years of age or older are eligible for inclusion. Residents of Wales will be defined through their identification in the Welsh Demographic Service Dataset (WDSD).

The NHS assigns each patient domiciled in the United Kingdom a unique number. This NHS number links across various NHS data systems. The encrypted and anonymized ALFs are derived from these NHS numbers. Therefore, patients attending ED whose identity cannot be connected to an NHS number (eg, overseas visitors and tourists) will not have a corresponding ALF and will, by necessity, be excluded from analyses.

Allocation

Intervention patients will be identified in the VPT data and will have attended intervention EDs (in Cardiff and Swansea), subject to the above inclusion criteria. Control patients will be identified in the EDDS, and ED attendance was not in an intervention ED.

Progression Criteria

This is a definitive study. As the primary focus of the study uses routinely collected data, which is available for analysis subject to information governance permissions and extraction, progression criteria are not applicable.

XSL•FO
RenderX

Sampling

From the intervention sites' data (Section 8.2.1), between October 2019 and December 2022, the Cardiff VPT contacted 2312 patients, of whom 77% accepted VPT support (Figure 1). We conservatively estimated that there will be 2500 patients that engaged with the Cardiff VPT across the 4 years (2019-2024) of VPT operation and a further 900 from the 2 years (2021-2024) operation in Swansea. Across the entirety of Wales, there are approximately 1 million ED attendances each year.

Figure 1. Count of patients, by month, identified by the Cardiff violence prevention team (VPT) as attending due to a violence-related injury, with the number that subsequently engaged with the VPT.

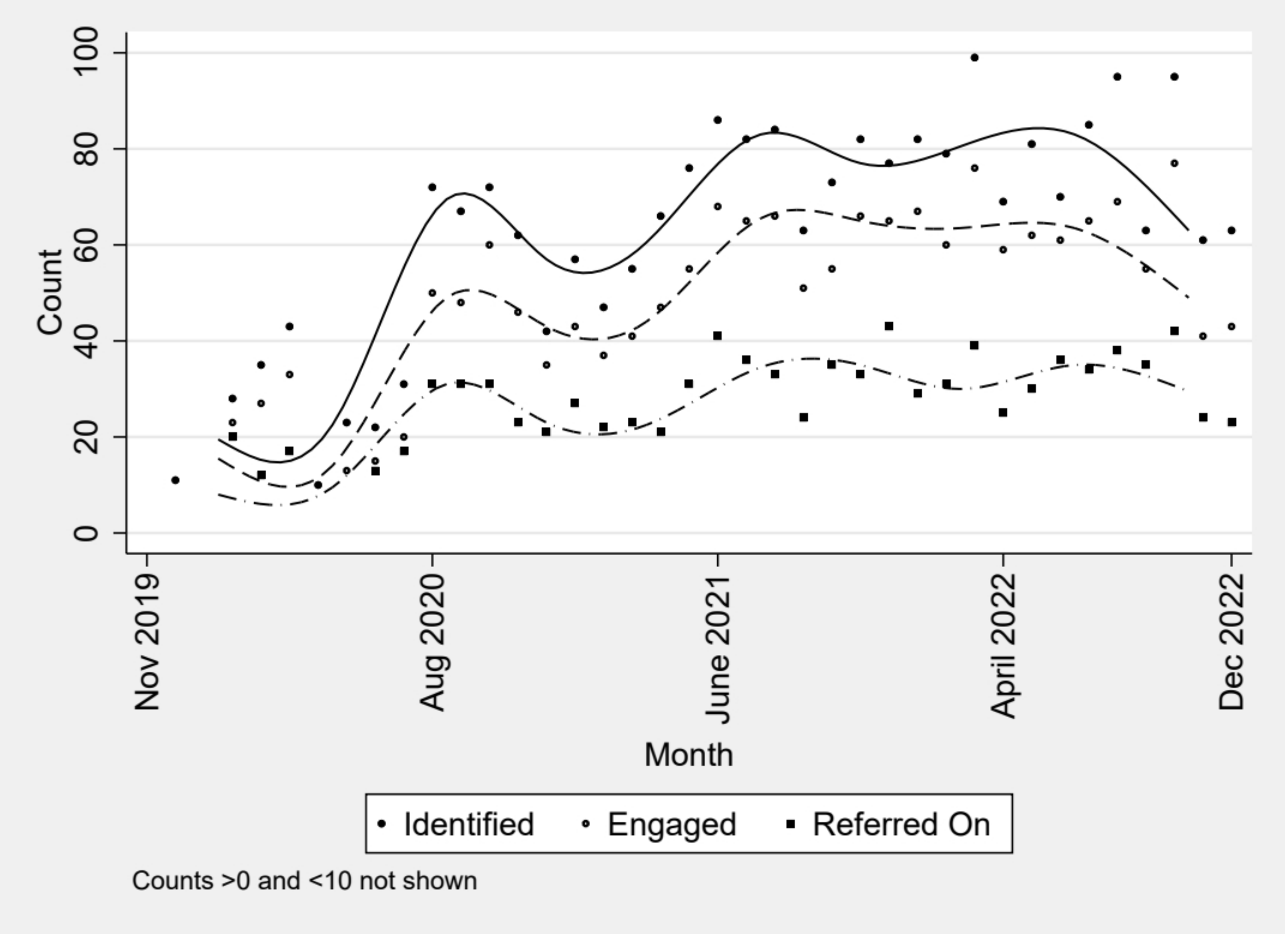

Initial estimates from Cardiff VPT suggest that 3% of those engaging with the VPT reattended ED at least once within one year, compared to 23% of patients who did not engage with the VPT. Data from 2015 and 2016 suggest that the frequency of unscheduled attendances for patients with at least one ARA (mean 2.35 attendances) is greater than for patients making an unscheduled attendance without evidence of an assault (mean attendances 1.73). For a simple Cox survival model, (α=.05; β=.90), and a hazard ratio of 0.8, a total N of 845 is required.

To realize the recurrent nature of analyses, simulation [44] (1000 estimates per point estimate) was used across varying follow-up periods, which suggests a 12-month follow-up period and total N of 300 is adequate to identify a significant effect. By increasing the number of controls, statistical power will be further enhanced [45].

Analytic Strategy

Objective 1: Effectiveness

Effectiveness Overview

Our primary outcome is unplanned ED attendance. It represents the cost to the NHS of serious health care events and acts as a proxy for events eliciting acute health care needs. EDs provide acute care for patients without prior appointment and the aftercare of patients who have received ED treatment, but where there is no alternative provision (eg, for out-of-area tourists). There can, therefore, be follow-up and planned appointments in ED. These appointments in ED will be made where, for example, there is an element of diagnostic uncertainty and a review is required in the ED context, or for patients where other follow-up arrangements are likely to fail (eg, visitors to the area without access to primary care) [46]. Follow-up and planned appointments in ED are typically a continuation of the initial unplanned attendance or referral from another health care provider and are not valid outcomes for EDVIPE, as they are not elicited in response to acute health care needs. Thus, for Objective 1 we will censor the timeline. Left-side censoring at

XSL•FO
RenderX

birth or when someone takes up residence in Wales. Right-side censoring when someone dies or moves out of Wales. We further interval censor the timeline to account for repeat ED attendances associated with a health event, such as a referral from a local emergency hospital to a Major Trauma Centre (MTC).

Discontinuous Risk Interval

Accounting for periods when individuals are not at risk is an essential consideration in repeated time-to-event models [47-50]. The clearest example in the current context is ensuring time at risk does not extend beyond the date of death or originate before birth. Similarly, time at risk will also be left-side censored if patients move into Wales and right-side censored if they move away from Wales. With no adjustment for these discontinuous risk intervals, the time at risk will be incorrect, increasing a greater likelihood of Type II errors.

How patients are routed through emergency care pathways in Wales also influences time at risk. ED attendances are mainly determined by the acuity of the patient's condition and the urgency with which they need to be seen. These decisions can be made by the Welsh Ambulance Service Trust (WAST), a MIU, or in the local ED. It is feasible that a patient initially attends a local ED to be stabilized, is assessed, and requires referral to a MTC or trauma unit (TU). Each MTC and TU is attached to an ED, and therefore, in response to severe injury, patients are registered in more than one ED if they are referred from an ED without trauma facilities to EDs that are attached to a TU or MTC.

In Wales, emergency care is provided in MIUs, EDs (Local Emergency Hospitals, and Rural Trauma Facilities), TUs, and MTCs. There is one MTC in Cardiff University Hospital Wales, which services South and West Wales and South Powys and acts as a TU for the local population. Morriston Hospital in Swansea is a TU, but with additional specialist services (eg, orthoplastics), meaning some patients otherwise destined for the MTC would be referred there instead. North Wales is serviced by the MTC in the Royal Stoke University Hospital in England.

Patient admission is described using spells and superspells. A spell represents patient care under a single specialty, and a superspell comprises multiple spells, where 2 spells are considered part of the same superspell if admission to the second specialty occurs within 48 hours of discharge from the first. ED attendances included within the same superspell (eg, transfer from an ED to an MTC) will therefore be attributable to the same initiating event, whether planned or unplanned. Therefore, only unscheduled attendances at the initiation of a superspell will be included, and additional ED attendances within the same superspell will be analytically censored. The duration of a superspell constitutes a discontinuity in a patient's exposure to risk, which is then accommodated in our analytic approach; this is described further in Figure 2.

Figure 2. A simplified example of discontinuous risk intervals as they relate to unscheduled Emergency Department (ED) attendance and superspells.

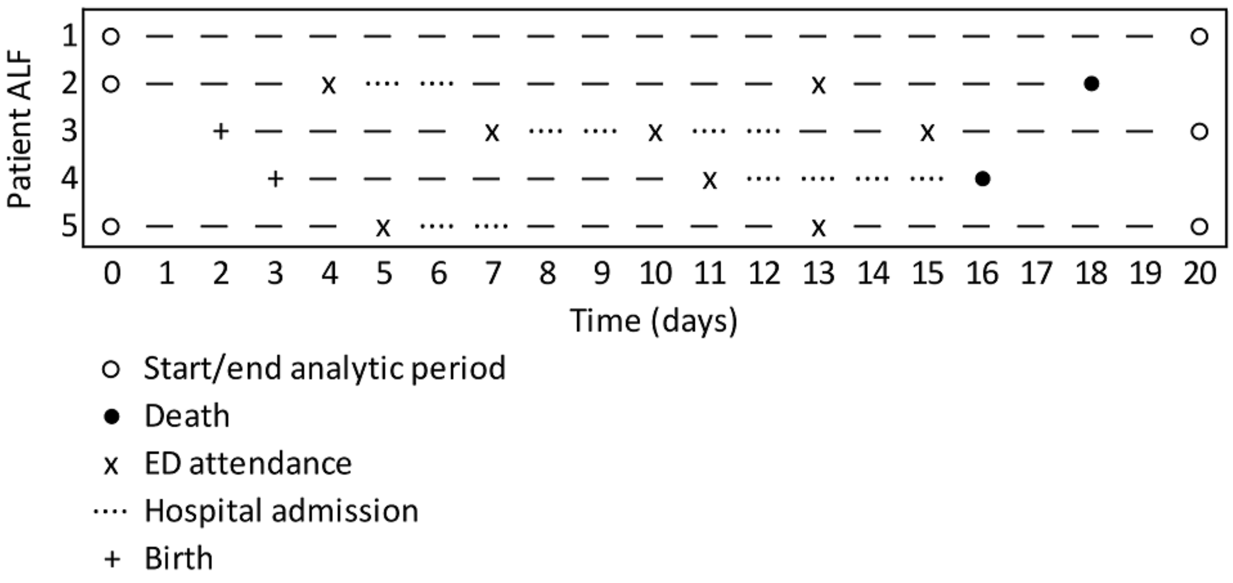

In Figure 2, a hypothetical analytic period runs from day 0 to day 20. Patient 1 lived in Wales for the duration of the study but had no ED attendances. Patient 2 lived in Wales at the start of the analytic period but died on day 18. They had 2 ED attendances, with the first resulting in a 2-day stay in the hospital. Their time at risk for the first ED attendance is 3 days, and for the second 6 days, with a total time at risk of 16 days.

Patient 3 was born in Wales on day 2, had 3 ED attendances, and was alive and living in Wales at the end of the analytic period. However, their second ED attendance (eg, transfer to an MTC) occurred in a superspell associated with the first ED attendance, and this is dropped from consideration. They therefore had 2 ED attendances, with the time at risk for the

XSL•FO
RenderX

first as 4 days and the time at risk for the second as 2 days. Their total time at risk is 10 days.

We assume that unscheduled ED attendances are independent and unordered, and therefore assume a common baseline hazard for all events. Thus, the hazard function, , for the kth ED event for each subject (*i*) is:

$$\lambda_{ik}(t) = \lambda_0(t)e^{x_{ik}\beta}$$

However, it is feasible that operational differences across Local Health Boards might influence the likelihood that ED attendance varies across groups of patients. For example, initiatives that influence the likelihood patients are conveyed into emergency care or alternative provision in the community for lower acuity patients. We introduce a random effect, , to describe variation in risk, or frailty, that can be introduced [51]:

$$\lambda_{ik}(t) = \lambda_0(t)\alpha_i e^{x_{ik}\beta}$$

Dates and Times

Records on the date and time of events in routine data require consideration. With most events recorded by day, we are obliged to use day as the primary measure of time, and therefore collisions will occur.

In EDDS the date and time of attendance and discharge are recorded, and several events can occur on the same day. For example, it is feasible that a patient attends ED on multiple occasions and on the same day. ED attendances on the same day are collapsed onto one another under the superspell assumption. In the WDSD, while date of birth is approximated to the Monday of the week in which they were born (their Week of Birth), date of death is accurate to that degree of granularity.

For example, a patient might attend ED at 9 AM and die at 2 PM on the same day. To avoid inconsistencies such as this patient entering the study cohort after death, we will adopt the usual approach and add a small value (ε) to dates to preserve the chronological order of events [52].

Matching

It is not possible to manipulate allocation to treatment, and we therefore rely on quasi-experimental methods to infer treatment effects. The purpose of matching control and intervention patients is to allow derivation of the average treatment effect under the assumption that allocation is not conditional on observed confounders. Matching on values of covariates between the treated and untreated groups avoids the bias introduced by covariates that influence the outcome variable. The goal being to find a subset of data that is closest to an exact match on observed covariates [53]. The primary analysis will be conducted using coarsened exact matching, with a minimum 1:1 ratio, with secondary analyses undertaken using propensity score matching.

For categorical covariates, exact matching is a sensible alternative. Here a match is considered acceptable only if the values of all covariates are equal between treated and untreated potential matches. Continuous covariates can, for the purpose of using exact matching, be turned into categorical covariates by defining category intervals; the resulting method is called coarsened exact matching. For matching, covariate categories can be grouped together to form larger (and fewer) categories, improving efficiency [53-55]. This approximates to a fully blocked experiment and requires temporarily coarsening variables.

Propensity score matching reduces the multidimensional space of covariates to a single summary covariate, the propensity score, typically derived using a logistic equation and performing regression on the treatment group for the covariates chosen to match with. The proximity of a potential match to the given subject is estimated in terms of the closeness of their propensity scores. This method simplifies subsequent analyses, as matching refers only to one variate, the propensity score. However, propensity score matching can increase, rather than decrease, the imbalance of the covariates in the samples [53]. A challenge is optimizing the trade-off between the quality of matches and the sample size. A larger sample reduces sampling error and can increase the study power, but including matches of lower quality may lead to greater residual imbalance [56]. The R package Matching Frontier attempts to address these issues by calculating the entire balance-sample size frontier, from which the user can easily choose one, several, or all subsamples to use for their final analysis, given their own choice of imbalance metric and quantity of interest [57,58].

Missing Data

Missing data may arise specific to the use of census data, where it is feasible that records are missing entirely or patients did not disclose personal characteristics. However, given that these characteristics are recorded across multiple datasets, the expectation is that missingness will minimally impact the analyses. Nevertheless, missingness will be considered should it arise in the process of analysis.

Objective 2: Ascertainment

The hypothesis is that a violence attendance is more likely to be ascertained as such with the additional VPT resource present in ED. All unplanned attendances can be coded as ARA or not (1, 0). The time series begins in January 2012, and the 2 sites (Cardiff and Swansea) can be individually compared with all other Type I EDs in Wales. This indicates that a difference-in-difference model [59] is appropriate. We will analyze Cardiff first, using the implementation in Swansea as a future replication of the intervention to facilitate a more robust causal interpretation of any effect. Additional descriptive analysis can explore patient groups (age, gender, and time of attendance) more likely to be ascertained by the VPTs. Unlike Objective 1, we are interested in all ED attendances, as the opportunity to provision safeguarding is applicable across all ED attendances, irrespective of the frequency of attendance.

Objective 3: Cost-Effectiveness Analysis

The aim of the economic evaluation is to understand the costs and consequences of providing a VPT versus no VPT within EDs. For this study, we will focus on the objectives of understanding whether the VPT represents value for money from an NHS perspective.

XSL•FO
RenderX

Methods

A within-study analysis will determine the short-term cost-effectiveness of offering VPT relative to not offering VPT within EDs. Estimates derived from this study will be used to inform a long-term decision analytic model examining the cost-effectiveness of providing, versus not providing, VPT within EDs over a longer time horizon. Alternative perspectives for the analysis will be considered, with the NHS perspective being the base case. Cost-effectiveness will be presented using standard statistics.

Exposure Variable

The exposure variable consists of patients engaging with the VPT.

Outcome Variables

Health-related outcome variables include improved mental health outcomes, measured as a reduction in primary and secondary care visits for a mental health reason, reduced substance use, improved physical health, measured as a reduction in ARAs in ED, and improved health-related quality of life (HRQoL), measured using injury-related estimates from the literature. Data on all outcomes will be sourced from the SAIL databank [43]. Estimates will be measured on a quarterly basis for the follow-up period (12-48 months). HRQoL will be estimated using the literature, in line with other studies on hospital-based violence prevention programs.

Within Study Statistical Analysis

Differences in overall mean outcomes will be analyzed using a fixed effects panel regression model, where we will control for individual and time-varying effects [60].

Covariates of Interest

The following covariates will be included within the fixed effects panel regression model, patient age, gender, severity of injury, number of years the VPT has been operating, year, and quarter. Referral uptake will be treated as a mediating variable and not adjusted for in the models.

Long-Term Cost-Effectiveness Analysis

A Markov decision model with quarterly cycles will be used to estimate the cost-effectiveness of offering VPTs in EDs. To determine cost-effectiveness using a health and social care perspective (primary analysis), the Markov model will be health-state based, with key health states likely to be healthy, injured, or dead. To support a multisector perspective (secondary analysis), the Markov model will be event-based.

Resource Use

Health care resource use will include number and type of visits with

- primary care (eg, General Practitioner),
- secondary care (eg, inpatient stays and outpatient clinic appointments), and
- other services (eg, other health care professional visits, physiotherapy, occupational therapy, mental health therapy, and drug and alcohol treatment).

Cost Data

Cost data will be estimated for health service use based on resource use from the SAIL databank with appropriate unit costs applied. Third sector resource use will be valued using cost data provided in the resource use questionnaires. Other unit cost estimates (eg, NHS reference costs, unit costs of health and social care) will be used to supplement where needed.

Differences in overall mean total health care costs (including primary and secondary care) between groups will be analyzed using generalized linear mixed models. Cost data are known to be left-skewed with a substantial number of zeroes, and there is no single dominant method for analysis [61]. We will test several potential models from the generalized linear mixed model family and identify the correct link function and distribution to use. Potential effect modifiers include age, gender, and deprivation. Mediating variables include intervention engagement. Potential confounding variables include age, gender, deprivation, and the number of years VPT has been operating. The regression model will also include time variables recording the year and quarter.

Costs and benefits will be discounted at 3.5% per annum as recommended by the HM Treasury [62].

Time Horizon

The time horizon for the decision analytic model will extend beyond the time frame of this study to encompass a period of time where, if possible, all relevant costs and outcomes for this appraisal have been accrued [63].

Cost-Effectiveness Analysis Outcomes

For the primary analysis, we will report the incremental cost-effectiveness ratio as the cost per quality-adjusted life-year, the net monetary benefit, and the net health benefit for those being offered versus not offered VPT assessment during their visit to the Accident and Emergency Department. The net monetary benefit and net health benefit will be assessed using a range of values (£15,000-£30,000 [approximately US $20,709-US $41,418, using an exchange rate of 1£≈1.38 US$] per quality-adjusted life year) representing a health decision maker's willingness-to-pay to obtain the distribution of net benefits at different levels of willingness-to-pay.

For the secondary (multisector) analysis, we report the cost per unit of effectiveness in natural units (eg, cost per reconviction avoided). We will also present the results using the extended impact inventory framework and consider alternative methods of aggregation [64].

Sensitivity Analysis

Parameter uncertainty will be assessed using a probabilistic sensitivity analysis, varying key parameters over a range of expected values, and running 1000 Monte Carlo simulations.

Social Costs

Additional exploratory analyses will estimate the broader social costs associated with the VPTs. Social costs will focus specifically on organization costs of supporting patients who have been referred to them by the VPTs. Cost data of organizations will be assessed using top-down gross costing.

XSL•FO
RenderX

Questionnaires or interviews, depending on interviewee preference, will be conducted with members of organizations to assess (1) the total annual expenditure cost for third sector and statutory organizations in the given year [65]. This cost is the total yearly cost of running the service, including costs of supporting patients, staff costs, and consumables. (2) The total number of users supported each year by the third sector and statutory organizations. (3) The percentage of those supported that were referred by the VPTs each year.

The given year will be 2022-2023; this is due to the Swansea team being implemented in January 2022. Top-down gross costing allows data to be aggregated and will allow us to estimate the mean cost of a "typical patient" to a "typical organization." We will include the distribution for each organization but will not attribute a specific cost to a specific organization. Furthermore, we will ask organizations for standard cost data (eg, cost per counseling session); this will be used as a comparison of our mean average. We will ask about waiting lists for support, which will be used to inform our knowledge.

PPIE

Extensive PPIE has been and will continue to be undertaken. The rationale is that many patients managed by the VPTs will be vulnerable, with some at the beginning of their journey in the support they receive. The expectations were that these patients would be unlikely to reflect meaningfully on the VPT within the study timeline and therefore alternative opportunities to explore patients' perceptions were required. Furthermore, follow-up qualitative work with young adults in emergency care, the dominant group in ED, requires considerable resourcing and suffers from high levels of attrition [66]. We therefore sought PPIE engagement in order that those with experience of the emergency health care system were able to feed into the project, co-produce methods, provide their interpretation of the results and assist with interpretation and dissemination. Groups include survivors of domestic violence, carers, and those who have experienced alcohol and drug dependence, homelessness, sexual exploitation, and mental health issues. One PPIE co-investigator was appointed to lead on monitoring equality and diversity, with a second, and experienced, PPIE co-investigator supporting inexperienced PPIE members. PPIE activity was captured using the short-form Guidance for Reporting Involvement of Patients and the Public (GRIPP) [67].

The PPIE groups include lay members with experience of PPIE work and service users from primary and emergency care research, who provided lay perspectives to the research team during the development and conduct of the study; subsequent engagement was undertaken to strengthen the relevance, quality, and dissemination of the research. Additional PPIE members were recruited. These members had lived experience relevant to the patients who are the subject of the intervention (homelessness and domestic violence). Service Users for Primary and Emergency Care Research (SUPER) and the PPIE co-investigators advised on how the research team should engage with those who have lived experience but had not had prior experience of PPIE involvement.

PPIE Activity

SUPER provided feedback on the original proposal and then provided advice on how the materials should be developed for the 2 less experienced PPIE groups. These PPIE groups with lived experience were recruited to give input on the protocol. One group consisted of people with lived experience of homelessness and related conditions, recruited from a charity supporting those who are experiencing homelessness. The second group was comprised of survivors of domestic violence and was recruited through Welsh Women's Aid. The PPIE group members gave feedback on the VPTs and provided their first impressions of the research protocol. The groups also helped to develop a list of potential organizations to disseminate research findings and discussed methods on how this could be achieved. They explained what may be going on in the lives of people who experience violence and where people might turn to for support.

PPIE Design Input

PPIE contributed in different ways to the initial development of the research proposal and the development of the research protocol (Textbox 3).

XSL•FO
RenderX

Textbox 3. Contributions of Patient and Public Involvement and Engagement (PPIE) to study design, protocol development, and data selection.

PPIE contributed to the development of the research proposal and protocol in the following ways:

- Initial consultation in planning the funding application.
- The research proposal was reviewed in July 2021, and the input and advice contributed to a successful funding application. The research team also responded to the formative comments when developing the protocol and continues to build on them.
- PPIE work influenced the types of data being included in the study. For example, an issue was raised about whether some patients would admit to experiencing violence in the Emergency Department (ED), and therefore opportunities for the Violence Prevention Teams to improve ascertainment.
- Data on ethnicity from the 2011 and 2021 Census, which will also have some indication of those residing in refugee centers (and nursing homes, hostels, etc), will be included in the study following recommendations that racial violence and the experiences of asylum seekers should be considered.
- It was further indicated that considering school attendance and exclusions data, free school meals and special educational needs would be valuable, in addition to educational attainment.
- The Emergency Department Violence Intervention Program Evaluation (EDVIPE) Stakeholder Reference Group now includes representation from primary care, secondary care, and the third sector, after working with these sectors was suggested.
- Following feedback, EDVIPE now involves PPIE groups with lived experience of different types of violence.
- Following consultation with PPIE lived experience groups, development and refinement of the protocol and the addition of exploratory work were undertaken to:
- Better understand the pathways patients follow up to their attendance in ED, notably whether General Practitioner consultations were not acted upon.
- To consider patient ascertainment and therefore eligibility for the intervention in ED, as some victims may not realize that they are victims of violence.
- Whether one or two nurses are sufficient to manage an expected high caseload of patients attending ED due to assault, and whether the lack of 24-hour VPT provision would mean some patients are missed.
- Some patients might choose to avoid the police and therefore be less reluctant to receive support from the police or police-aligned services. We might explore this in any VPT referral data available.
- There were concerns that intervention-related activity might become known to the perpetrator, therefore elevating the risk of subsequent harm. We can consider the immediacy of postintervention reattendance by patient group.
- That there are unique challenges for those who are disabled, both in terms of ability to engage and the nature of the support required.
- Some patients, notably those with children, may be less willing to engage, as they would not want to risk losing their home or children. We can consider engagement by gender and presence of dependent children in the patient’s residence.
- The judicial system emphasizes the right for both parents to be involved with their children, if any. Involvement of parents in the court system might be associated with a blunted intervention effect.
- That PPIE involvement in future diffusion and dissemination activity would lend credence to the project’s communication strategy.

Ethical Considerations

The approval to access and analyze data housed in the SAIL Databank [43], an ISO (International Organization for Standardization) 27001 certified and UK Statistics Authority accredited secure data environment, was granted by the SAIL Independent Information Governance Review Panel (IGRP; Ref: 1421). The IGRP comprises representatives from various organizations and sectors, including the British Medical Association, Welsh Government, Public Health Wales, National Research Ethics Service, Digital Health and Care Wales (DHCW), Swansea Bay University Health Board, and members of the public. All routinely collected anonymized data held in SAIL are exempt from consent due to the anonymized nature of the databank (Section 251, Control of Patient Information; 2006 National Health Service Act). At no time will identifiable data be made available to the research team. ED staff will curate data pertaining to patients’ exposure to the intervention, which will be passed to DHCW, where it will be anonymized, and a project-specific ALF added, as will a residential ALF. These data will be passed to SAIL for linkage to anonymized VPT clinical data.

Results

As this is a protocol, there are no results. The study was funded in November 2021, preregistered in June 2022 (ISRCTN 41868) and commenced in November 2023. The current version of the protocol (v4.1) was published by the funders in July 2024 [68]. The collection of routine data used in the current study has no end date, as it is primarily generated by patient interactions with health services. The analyses for Objective 1 were completed in August 2025 with the expectation they will be publicly available in June 2026. The analyses for Objective 2 were completed in July 2025 and are published [69]. The analyses for Objective 3 were completed in January 2026 and are expected to be available in August 2026.

XSL•FO
RenderX

Discussion

Principal Findings

We hypothesize that, for Objective 1, we will find a reduction in ED attendance. Objective 2 is exploratory and arose through PPIE; while we expect that some patients may not be willing to disclose their exposure to violence, the extent of this effect in ED is not known. We hypothesize that the intervention will be cost-effective over a 10-year time horizon and from the perspective of the NHS.

As far as we are aware, this project will not only provide the first robust evaluation of violence prevention activities in ED, but the data available will also provide an unprecedented opportunity to consider some of the pathways that patients follow before, during, and after their attendance in ED. Ongoing PPIE and stakeholder engagement have already shaped the study objectives and methods. However, we are particularly interested in exploring some of the characteristics of patients that expose them to violence. There is growing interest in neurodevelopment conditions [70], and it is feasible that secondary analyses could inform the future design of ED-based violence prevention initiatives. Furthermore, there is a paucity of information on the costs and health consequences of violence. The primary outcomes from this effectiveness and cost-effectiveness evaluation will offer novel insights to the benefit of future research and therefore violence prevention. We anticipate future research might therefore focus on how intersectional vulnerabilities, such as neurodevelopmental conditions, age, gender and ethnicity, and how they interact with residential deprivation to influence the likelihood of serious violence.

Strengths and Limitations

Linkage requires knowledge of the patients' identity, anonymized and encoded as ALFs, to be included in the data return. There are instances where individuals may prefer to remain anonymous. Analyses are therefore limited to those who can be identified in routine data and linked to the WDS and therefore EDDS and related datasets. It is feasible that some may attempt to conceal their identity, and this could correspond to greater vulnerability. Furthermore, our reliance on administrative data means there is no information on the context in which their exposure to violence arose. There may be a marked difference in the modifiable risks and vulnerabilities for patients sustaining injury in and around premises licensed for the sale and onsite consumption of alcohol and those experienced criminal or sexual exploitation. Conversely, our primary hypothesis is that the additionality of the VPT intervention teams means ED is better able to work with patients and therefore address the variation of patients that attend.

Dissemination, Outputs, and Anticipated Impact

A diffusion and dissemination plan will be co-produced with stakeholders and PPIE groups. In so doing, we will define audiences (policy, practitioner, academic, lay, etc) that might find the project outcomes of interest. In so doing, we will determine appropriate modalities to communicate with each, including presentations, briefing documents, and media events, with content informed by audience need.

PPIE, Policy, and Practitioner Focused

An ongoing evaluation of VRUs and VPUs is to recommend that greater attention is paid to evidence-based interventions and that activities should consider the possible involvement of schools and therefore the Department for Education. Education data has been included, and we can therefore contribute to the evidence-based linking of school activity to violence. In addition, our PPIE engagement highlighted the need to consider ARA predictors, and engagement with the Home Office will further identify intermediate outcomes. We therefore aim to undertake interim analyses that will inform the final analysis and respond to significant emerging policy questions as they relate to evidence-based violence prevention and reduction initiatives. While these will likely translate to academic papers, we also seek to produce more accessible outputs and exploit existing networks in that respect.

Conclusion

This protocol describes, in detail, novel methods to undertake a novel and robust effectiveness and cost-effectiveness of Hospital Violence Intervention Programs. Engagement with the public and patients, including those with lived and living experience of violence, together with broad stakeholder engagement, has greatly influenced the study design and methods. For example, it was through patient engagement we became aware that not all patients might disclose their exposure to violence, an exploration of which is now a primary outcome. Furthermore, stakeholders being able to comment on the design of emergency care systems resulted in an analytic need to account for interval censoring in patient timelines. It is feasible that the novelty of this work will identify further matters that might influence the design of subsequent studies.

Acknowledgments

The authors would like to acknowledge Nurses Vicky Lee and Sarah Wilcox, leads for the intervention sites, for their support of this evaluation. Furthermore, we would like to thank the Public, Patient Involvement participants, many of whom had not prior experience working with researchers. Their enthusiasm and insights greatly impacted on this evaluation and the broader research team. Generative Artificial Intelligence was not used in any aspect of this work.

A Study Steering Committee (SSC) was convened to oversee and advise on progress. The committee included the Data Monitoring and Ethics functions and oversees any protocol amendments

Data Availability

The datasets to be analyzed during this study are available in the SAIL Databank repository [43].

XSL•FO
RenderX

Funding

This study is funded by the NIHR (Public Health Research Program [NIHR134055]). The views expressed are those of the authors and not necessarily those of the NIHR or the Department of Health and Social Care.
The funder had no role in the design of the study, and will not have any role during its execution, analyses, interpretation of the data, or decision to submit results.

Authors' Contributions

SCM, S Brophy, A Watkins, S Walker, A Battaglia, HY, TL, and D O'Reilly contributed to conceptualization, funding acquisition, and writing of the original draft and review and editing. SCM, S Brophy, A Watkins, and S Walker also contributed to investigation, methodology, project administration, and supervision, with SCM and S Walker additionally contributing to resources and visualization. A Bandyopadhyay and S Premji contributed to data curation, formal analysis, investigation, validation, and writing-review and editing. S Borgia and MH contributed to data curation and investigation. A Newbury contributed to investigation and writing-review and editing. D Rawlinson, LS, JS, and VS contributed to funding acquisition, with LS, JS, and VS also contributing to writing-review and editing.

Conflicts of Interest

None declared.

Multimedia Appendix 1

TIDieR checklist.
[DOCX File , 20 KB-Multimedia Appendix 1]

Multimedia Appendix 2

Confirmation of NIHR review and funding. NIHR: National Institute for Health and Care Research.
[PDF File (Adobe PDF File), 115 KB-Multimedia Appendix 2]

Multimedia Appendix 3

Confirmation of NIHR review and funding. NIHR: National Institute for Health and Care Research.
[PDF File (Adobe PDF File), 142 KB-Multimedia Appendix 3]

Multimedia Appendix 4

Peer review report by Public Health Research Program (PHR) Funding Committee, NIHR Evaluation, Trials and Studies Coordinating Centre (NETSCC), National Institute for Health Research (NIHR).
[PDF File (Adobe PDF File), 246 KB-Multimedia Appendix 4]

References

1. Purtle J, Dicker R, Cooper C, Corbin T, Greene M, Marks A, et al. Hospital-based violence intervention programs save lives and money. J Trauma Acute Care Surg. 2013;75(2):331-333. [doi: 10.1097/TA.0b013e318294f518] [Medline: 23887566]
2. Cunningham R, Knox L, Fein J, Harrison S, Frisch K, Walton M, et al. Before and after the trauma bay: the prevention of violent injury among youth. Ann Emerg Med. Apr 2009;53(4):490-500. [doi: 10.1016/j.annemergmed.2008.11.014] [Medline: 19162376]
3. Kaufman E, Rising K, Wiebe DJ, Ebler DJ, Crandall ML, Delgado MK. Recurrent violent injury: magnitude, risk factors, and opportunities for intervention from a statewide analysis. Am J Emerg Med. 2016;34(9):1823-1830. [FREE Full text] [doi: 10.1016/j.ajem.2016.06.051] [Medline: 27460511]
4. Kao AM, Schlosser KA, Arnold MR, Kasten KR, Colavita PD, Davis BR, et al. Trauma recidivism and mortality following violent injuries in young adults. J Surg Res. 2019;237:140-147. [doi: 10.1016/j.jss.2018.09.006] [Medline: 30914191]
5. Newton AS, Hartling L, Soleimani A, Kirkland S, Dyson MP, Cappelli M. A systematic review of management strategies for children's mental health care in the emergency department: update on evidence and recommendations for clinical practice and research. Emerg Med J. 2017;34(6):376-384. [doi: 10.1136/emermed-2016-205939] [Medline: 28119350]
6. Hinsliff-Smith K, McGarry J. Understanding management and support for domestic violence and abuse within emergency departments: a systematic literature review from 2000-2015. J Clin Nurs. 2017;26(23-24):4013-4027. [doi: 10.1111/jocn.13849] [Medline: 28403521]
7. Ansari S, Boyle A. Emergency department-based interventions for women suffering domestic abuse: a critical literature review. Eur J Emerg Med. 2017;24(1):13-18. [doi: 10.1097/MEJ.0000000000000416] [Medline: 27467558]

XSL•FO
RenderX

8. Brice JM, Boyle AA. Are ED-based violence intervention programmes effective in reducing revictimisation and perpetration in victims of violence? A systematic review. Emerg Med J. 2020;37(8):489-495. [doi: 10.1136/emermed-2019-208970] [Medline: 32554747]
9. Koenig KL, Benjamin SB, Beÿ CK, Dickinson S, Shores M. Emergency department management of the sexual assault victim in the COVID Era: a model SAFET-I guideline from San Diego county. J Emerg Med. 2020;59(6):964-974. [FREE Full text] [doi: 10.1016/j.jemermed.2020.07.047] [Medline: 32951933]
10. Goodall C, Jameson J, Lowe DJ. Navigator: A Tale of Two Cities. Glasgow, Scotland. Violence Reduction Unit; 2017.
11. Sivarajasingam V, Guan B, Page N, Peel E, Moore S, Shepherd J. Violence in England and Wales in 2019. Cardiff, UK. Cardiff University; 2020.
12. Sivarajasingam V, Guan B, Page N, Moore S, Shepherd J. Violence in England and Wales in 2020. Cardiff, UK. Cardiff University; 2021.
13. Introducing public health measures impact assessment 2019 (accessible version). GOV.UK. 2019. URL: https://tinyurl.com/3muws8bp [accessed 2026-01-23]
14. Shepherd JP, Ali MA, Hughes AO, Levers BG. Trends in urban violence: a comparison of accident department and police records. J R Soc Med. 1993;86(2):87-88. [FREE Full text] [Medline: 8433313]
15. Sutherland I, Sivarajasingam V, Shepherd JP. Recording of community violence by medical and police services. Inj Prev. 2002;8(3):246-247. [FREE Full text] [doi: 10.1136/ip.8.3.246] [Medline: 12226126]
16. Gray BJ, Barton ER, Davies AR, Long SJ, Roderick J, Bellis MA. A shared data approach more accurately represents the rates and patterns of violence with injury assaults. J Epidemiol Community Health. 2017;71(12):1218-1224. [FREE Full text] [doi: 10.1136/jech-2017-209872] [Medline: 29056592]
17. Bath R. A Whole-System Multi-Agency Approach to Serious Violence Prevention: A Resource for Local System Leaders in England. London, UK. Public Health England; 2019.
18. Serious violence strategy. GOV.UK. 2018. URL: https://www.gov.uk/government/publications/serious-violence-strategy [accessed 2018-01-23]
19. Violence Reduction Unit interim guidance. GOV.UK. 2020. URL: https://assets.publishing.service.gov.uk/media/5e7de2cfe90e0706ef2db559/12VRU_Interim_Guidance_FINAL__003_2732020.pdf [accessed 2026-01-23]
20. Fit for the Future: 10 Year Health Plan for England. NHS. 2019. URL: https://www.longtermplan.nhs.uk/ [accessed 2026-01-23]
21. Better care for people with co-occurring mental health and alcohol/drug use conditions. Public Health England. 2017. URL: https://assets.publishing.service.gov.uk/media/5a75b781ed915d6faf2b5276/Co-occurring_mental_health_and_alcohol_drug_use_conditions.pdf [accessed 2026-01-23]
22. Duke AA, Smith KMZ, Oberleitner LMS, Westphal A, McKee SA. Alcohol, drugs, and violence: a meta-meta-analysis. Psychol Violence. 2018;8(2):238-249. [doi: 10.1037/vio0000106]
23. Babor TF, Berglas S, Mendelson JH, Ellingboe J, Miller K. Alcohol, affect, and the disinhibition of verbal behavior. Psychopharmacology (Berl). 1983;80(1):53-60. [doi: 10.1007/BF00427496] [Medline: 6408672]
24. Malik NS, Munoz B, de Courcey C, Imran R, Lee KC, Chernbumroong S, et al. Violence-related knife injuries in a UK city; epidemiology and impact on secondary care resources. EClinicalMedicine. 2020;20:100296. [FREE Full text] [doi: 10.1016/j.eclinm.2020.100296] [Medline: 32300742]
25. Pallett J, Sutherland E, Glucksman E, Tunnicliff M, Keep J. A cross-sectional study of knife injuries at a London major trauma centre. Ann R Coll Surg Engl. 2014;96(1):23-26. [FREE Full text] [doi: 10.1308/003588414X13824511649616] [Medline: 24417825]
26. Boyle A, Frith C, Edgcumbe D, McDougall C. What factors are associated with repeated domestic assault in patients attending an emergency department? A cohort study. Emerg Med J. 2010;27(3):203-206. [doi: 10.1136/emj.2009.072033] [Medline: 20304886]
27. DeWall CN, Anderson CA, Bushman BJ. The general aggression model. Psychology of Violence. 2011;2(3):245-258. [doi: doi: 10.1037/a0023842]
28. Allen JJ, Anderson CA. General aggression model. In: The International Encyclopedia of Media Effects. Hoboken, NJ. Wiley; 2017:1-15.
29. Farrington DP. Early predictors of adolescent aggression and adult violence. Violence Vict. 1989;4(2):79-100. [doi: 10.1891/0886-6708.4.2.79]
30. Hawkins JD, Herrenkohl T, Farrington DP, Brewer D, Catalano RF, Harachi TW. A review of predictors of youth violence. In: Howell JC, editor. Serious and Violent Juvenile Offenders: Risk Factors and Successful Interventions. Thousand Oaks, CA. Sage Publications, Inc; 1998:106-146.
31. Piquero AR, Jennings WG, Diamond B, Reingle JM. A systematic review of age, sex, ethnicity, and race as predictors of violent recidivism. Int J Offender Ther Comp Criminol. 2015;59(1):5-26. [doi: 10.1177/0306624X13514733] [Medline: 24335783]
32. WHO. World Report on Violence and Health. Geneva. World Health Organisation; 2002.

XSL•FO
RenderX

33. Long SJ, Fone D, Gartner A, Bellis MA. Demographic and socioeconomic inequalities in the risk of emergency hospital admission for violence: cross-sectional analysis of a national database in Wales. BMJ Open. 2016;6(8):e011169. [FREE Full text] [doi: 10.1136/bmjopen-2016-011169] [Medline: 27558900]
34. Ostrowsky MK. The social psychology of alcohol use and violent behavior among sports spectators. Aggress Violent Behav. 2014;19(4):303-310. [doi: 10.1016/j.avb.2014.05.001]
35. Fazel S, Gulati G, Linsell L, Geddes JR, Grann M. Schizophrenia and violence: systematic review and meta-analysis. PLoS Med. 2009;6(8):e1000120. [FREE Full text] [doi: 10.1371/journal.pmed.1000120] [Medline: 19668362]
36. Fu F, Nowak MA, Christakis NA, Fowler JH. The evolution of homophily. Sci Rep. 2012;2:845. [FREE Full text] [doi: 10.1038/srep00845] [Medline: 23150792]
37. Moore S, Van Godwin J, Moore G, Hamilton M, O'Reilly D. Practitioner experiences of developing and implementing two UK ED-based hospital violence intervention programmes: a process evaluation. Emergency Medicine Journal. 2025;42:536-541. [doi: 10.1136/emermed-2024-214333]
38. Hoffmann TC, Glasziou PP, Boutron I, Milne R, Perera R, Moher D, et al. et al. Better reporting of interventions: template for intervention description and replication (TIDieR) checklist and guide. BMJ. Mar 07, 2014;348:g1687. [FREE Full text] [doi: 10.1136/bmj.g1687] [Medline: 24609605]
39. Florence C, Shepherd J, Brennan I, Simon T. Effectiveness of anonymised information sharing and use in health service, police, and local government partnership for preventing violence related injury: experimental study and time series analysis. BMJ. 2011;342:d3313. [FREE Full text] [doi: 10.1136/bmj.d3313] [Medline: 21680632]
40. Linden A. A matching framework to improve causal inference in interrupted time-series analysis. J Eval Clin Pract. 2018;24(2):408-415. [doi: 10.1111/jep.12874] [Medline: 29266646]
41. Linden A, Yarnold PR. Using machine learning to evaluate treatment effects in multiple-group interrupted time series analysis. J Eval Clin Pract. 2018;24(4):740-744. [doi: 10.1111/jep.12966] [Medline: 29888469]
42. Evaluation of Violence Reduction Units 2020/21. URL: https://tinyurl.com/38pvnwbt [accessed 2026-01-23]
43. Jones KH, Ford DV, Thompson S, Lyons R. A profile of the SAIL databank on the UK secure research platform. Int J Popul Data Sci. 2019;4(2):1134. [FREE Full text] [doi: 10.23889/ijpds.v4i2.1134] [Medline: 34095541]
44. Feiveson AH. Power by simulation. Stata J. 2002;2(2):107-124. [doi: 10.1177/1536867x0200200201]
45. Hennessy S, Bilker WB, Berlin JA, Strom BL. Factors influencing the optimal control-to-case ratio in matched case-control studies. Am J Epidemiol. 1999;149(2):195-197. [doi: 10.1093/oxfordjournals.aje.a009786] [Medline: 9921965]
46. Royal College of Emergency Medicine. Emergency Department Follow-up Clinics. London.; 2015. URL: https://tinyurl.com/3skzwd6b
47. Law CG, Brookmeyer R. Effects of mid-point imputation on the analysis of doubly censored data. Stat Med. 1992;11(12):1569-1578. [doi: 10.1002/sim.4780111204] [Medline: 1439361]
48. Rücker G, Messerer D. Remission duration: an example of interval-censored observations. Stat Med. 1988;7(11):1139-1145. [doi: 10.1002/sim.4780071106] [Medline: 3201039]
49. Turnbull BW. The empirical distribution function with arbitrarily grouped, censored and truncated data. J R Stat Soc Ser B (Methodol). 1976;38(3):290-295. [doi: 10.1111/j.2517-6161.1976.tb01597.x]
50. Guo Z, Gill TM, Allore HG. Modeling repeated time-to-event health conditions with discontinuous risk intervals. Methods Inf Med. 2018;47(02):107-116. [doi: 10.3414/me0478]
51. Balan TA, Putter H. A tutorial on frailty models. Stat Methods Med Res. 2020;29(11):3424-3454. [doi: 10.1177/0962280220921889] [Medline: 32466712]
52. Gould W. Why can’t a subject enter and die at the same time in the Cox model? Stata FAQs. Texas. StataCorp URL: https://www.stata.com/support/faqs/statistics/time-and-cox-model/
53. King G, Nielsen R. Why propensity scores should not be used for matching. Polit Anal. 2019;27(4):435-454. [doi: 10.1017/pan.2019.11]
54. King G, Nielsen R, Coberley C, Pope JE, Wells A. Comparative effectiveness of matching methods for causal inference. In: Institute for Quantitative Social Science. Cambridge. Harvard University; 2011.
55. Stuart EA. Matching methods for causal inference: a review and a look forward. Stat Sci. 2010;25(1):1-21. [FREE Full text] [doi: 10.1214/09-STS313] [Medline: 20871802]
56. Imbens GW. Matching methods in practice: Three examples. J Human Resources. 2015;50(2):373-419. [doi: 10.3368/jhr.50.2.373]
57. King G, Lucas C, Nielsen RA. Matching frontier. Harvard University. 2017. URL: https://projects.iq.harvard.edu/frontier [accessed 2026-01-23]
58. King G, Lucas C, Nielsen RA. The balance - sample size frontier in matching methods for causal inference. Am J Polit Sci. 2016;61(2):473-489. [doi: 10.1111/ajps.12272]
59. Wing C, Simon K, Bello-Gomez R. Designing difference in difference studies: best practices for public health policy research. Annu Rev Public Health. 2018;39:453-469. [FREE Full text] [doi: 10.1146/annurev-publhealth-040617-013507] [Medline: 29328877]
60. Jones AM. Panel data methods and applications to health economics. In: Palgrave Handbook of Econometrics. Cham. Springer; 2007:557-631.

XSL•FO
RenderX

61. Jones AM, Lomas J, Rice N. Healthcare cost regressions: going beyond the mean to estimate the full distribution. Health Econ. 2015;24(9):1192-1212. [FREE Full text] [doi: 10.1002/hec.3178] [Medline: 25929525]
62. The green book: central government guidance on appraisal and evaluation. GOV.UK. 2018. URL: https://www.gov.uk/government/publications/the-green-book-appraisal-and-evaluation-in-central-government [accessed 2026-11-23]
63. Drummond MF, Sculpher MJ, Claxton K, Stoddart GL, Torrance GW. Methods for the Economic Evaluation of Health Care Programmes. Oxford. Oxford University Press; 2015.
64. Walker S, Griffin S, Asaria M, Tsuchiya A, Sculpher M. Striving for a societal perspective: a framework for economic evaluations when costs and effects fall on multiple sectors and decision makers. Appl Health Econ Health Policy. 2019;17(5):577-590. [FREE Full text] [doi: 10.1007/s40258-019-00481-8] [Medline: 31098947]
65. Špacírová Z, Epstein D, García-Mochón L, Rovira J, Olry de Labry Lima A, Espín J. A general framework for classifying costing methods for economic evaluation of health care. Eur J Health Econ. 2020;21(4):529-542. [FREE Full text] [doi: 10.1007/s10198-019-01157-9] [Medline: 31960181]
66. Irving A, Buykx P, Amos Y, Goodacre S, Moore SC, O'Cathain A. The acceptability of alcohol intoxication management services to users: a mixed methods study. Drug Alcohol Rev. 2020;39(1):36-43. [FREE Full text] [doi: 10.1111/dar.13002] [Medline: 31769571]
67. Staniszewska S, Brett J, Simera I, Seers K, Mockford C, Goodlad S, et al. GRIPP2 reporting checklists: tools to improve reporting of patient and public involvement in research. BMJ. 2017;358:j3453. [FREE Full text] [doi: 10.1136/bmj.j3453] [Medline: 28768629]
68. Moore SC, Brophy S, Bandyopadhyay A, Newbury A, Hamilton M, Battaglia A. Protocol: A Quasi-Experimental Effectiveness and Cost-Effectiveness Evaluation of Emergency Department Violence Intervention Programmes in the United Kingdom Southampton. NIHR; 2024. URL: https://njl-admin.nihr.ac.uk/document/download/2047009 [accessed 2026-01-23]
69. Hamilton M, Snowdon L, Shepherd J, Premji S, Battaglia A, Yeomans H, et al. Nurse-led hospital violence intervention programmes improve emergency department identification of violence-related visits. Am J Emerg Med. 2026;99:376-380. [FREE Full text] [doi: 10.1016/j.ajem.2025.10.040] [Medline: 41175441]
70. Whiting D, Lichtenstein P, Fazel S. Violence and mental disorders: a structured review of associations by individual diagnoses, risk factors, and risk assessment. Lancet Psychiatry. 2021;8(2):150-161. [doi: 10.1016/S2215-0366(20)30262-5] [Medline: 33096045]

Abbreviations

ALF: Anonymous Linkage Field
ARA: Assault-Related Attendance
DHCW: Digital Health and Care Wales
ED: Emergency Department
EDDS: Emergency Department Data Set
EDVIPE: Emergency Department Violence Intervention Program Evaluation
GRIPP: Guidance for Reporting Involvement of Patients and the Public
HVIP: Hospital-Based Violence Intervention Program
IDVA: Independent Domestic Violence Advocate
IGRP: Independent Information Governance Review Panel
ISO: International Organization for Standardization
ISVA: Independent Sexual Violence Advisor
MARAC: multiagency risk assessment conference
MIU: minor injury unit
MTC: Major Trauma Centre
NHS: National Health Service
PMS: Patient Management System
PPIE: public patient involvement and engagement
SAIL: Secure Anonymised Information Linkage
SUPER: Service Users for Primary and Emergency Care Research
TIDieR: Template for Intervention Description and Replication
TU: Trauma Unit
VPT: violence prevention team
VPU: violence prevention unit
VRU: Violence Reduction Unit
WAST: Welsh Ambulance Service Trust
WDSD: Welsh Demographic Service Database
WSMA: whole system multiagency

XSL•FO
RenderX

YEF: Youth Endowment Fund

Edited by J Sarvestan; The proposal for this study was peer reviewed by the Public Health Research Program (PHR) Funding Committee, NIHR Evaluation, Trials and Studies Coordinating Centre (NETSCC), National Institute for Health Research (NIHR). See the Multimedia Appendix for the peer-review report; Submitted 21.Oct.2025; accepted 19.Jan.2026; published 18.Feb.2026.

Please cite as:
Moore S, Brophy S, Bandyopadhyay A, Newbury A, Hamilton M, Battaglia A, Lowe T, O'Reilly D, Rawlinson D, Snowdon L, Shepherd J, Sivarajasingam V, Watkins A, Walker S, Premji S, Borgia S, Yeomans H
Effectiveness and Cost-Effectiveness of Emergency Department–Based Violence Intervention Programs in the United Kingdom: Protocol for a Quasi-Experimental Study
JMIR Res Protoc 2026;15:e86247
URL: https://www.researchprotocols.org/2026/1/e86247
doi: 10.2196/86247
PMID:

©Simon Moore, Sinead Brophy, Amrita Bandyopadhyay, Annemarie Newbury, Megan Hamilton, Adele Battaglia, Trudy Lowe, David O'Reilly, David Rawlinson, Lara Snowdon, Jonathan Shepherd, Vaseekaran Sivarajasingam, Alan Watkins, Simon Walker, Shainur Premji, Sophie Borgia, Henry Yeomans. Originally published in JMIR Research Protocols (https://www.researchprotocols.org), 18.Feb.2026. This is an open-access article distributed under the terms of the Creative Commons Attribution License (https://creativecommons.org/licenses/by/4.0/), which permits unrestricted use, distribution, and reproduction in any medium, provided the original work, first published in JMIR Research Protocols, is properly cited. The complete bibliographic information, a link to the original publication on https://www.researchprotocols.org, as well as this copyright and license information must be included.

XSL•FO
RenderX
